# Rapid and reversible optical switching of cell membrane area by an amphiphilic azobenzene

**DOI:** 10.1038/s41467-023-39032-0

**Published:** 2023-06-23

**Authors:** Fabian Höglsperger, Bart E. Vos, Arne D. Hofemeier, Maximilian D. Seyfried, Bastian Stövesand, Azadeh Alavizargar, Leon Topp, Andreas Heuer, Timo Betz, Bart Jan Ravoo

**Affiliations:** 1 Organic Chemistry Institute, University of Münster, Münster, Germany; 2 Center for Soft Nanoscience, University of Münster, Münster, Germany; 3 Third Institute of Physics—Biophysics, University of Göttingen, Göttingen, Germany; 4 Institute of Physical Chemistry, University of Münster, Münster, Germany

**Keywords:** Supramolecular chemistry, Membrane biophysics, Biophysical chemistry, Small molecules

## Abstract

Cellular membrane area is a key parameter for any living cell that is tightly regulated to avoid membrane damage. Changes in area-to-volume ratio are known to be critical for cell shape, but are mostly investigated by changing the cell volume via osmotic shocks. In turn, many important questions relating to cellular shape, membrane tension homeostasis and local membrane area cannot be easily addressed because experimental tools for controlled modulation of cell membrane area are lacking. Here we show that photoswitching an amphiphilic azobenzene can trigger its intercalation into the plasma membrane of various mammalian cells ranging from erythrocytes to myoblasts and cancer cells. The photoisomerization leads to a rapid (250-500 ms) and highly reversible membrane area change (ca 2 % for erythrocytes) that triggers a dramatic shape modulation of living cells.

## Abstract (web summary)

The surface area of living cells is tightly regulated. Here, authors show that the surface area and shape of cells can be reversibly manipulated with light using amphiphilic molecular photoswitches that enter and leave the membrane.

## Introduction

Lipid bilayer membranes are a central part of cellular architecture and both their molecular organization and total area are tightly regulated by processes such as endocytosis and exocytosis, clathrin coated pits and caveolae, but also mechanical interaction with the underlying cytoskeleton^1–4^. Furthermore, the membrane area is directly influencing membrane tension which has become a subject of major studies in the past year as it was related to fundamental cell biological processes such as cell differentiation^5,6^, cell migration in cancer and immune response^7–9^ and cell volume homeostasis^10^. The obvious importance of membrane area control is unfortunately contrasted by a lack of simple tools to manipulate the membrane area. While micropipette aspiration techniques enable the manipulation of membrane tension, and membrane tube pulling experiments may read out general properties of the membrane, adding or removing lipids in a controlled and rapid way remains difficult^11–14^. From an experimental point of view, it would be desirable to have the possibility to not only locally alter the membrane area, but to also do this in a well-timed fashion, ideally without directly intervening into the system to simplify the experimental access. In this line of reasoning, an optical and reversible methodology can be seen as optimal as it would meet all of the above-mentioned requirements. Although a previous approach using a hydrophobic *ortho*-tetrafluoro-azobenzene has demonstrated that such an optical trigger to change membrane area is possible, the method suffered from slow temporal control on the range of minutes. Additionally, these hydrophobic photoswitches were not applied to membranes of living cells^15^.

Besides the overall experimental urge to obtain photo induced access to manipulate the membrane area, a simple and relevant living model system is required to demonstrate the feasibility of such a photoswitch. Here red blood cells (RBCs) are a highly suitable target cell, as their shape is well controlled, and even small changes in surface-to-volume ratio are known to trigger drastic shape changes^16^. Besides these experimental advantages, RBC shape stability is still not fully understood. This is caused by the complex interaction of mechanical forces and elastic contributions from the membrane and the underlying cytoskeleton^17,18^. In contrast, it is well established that for optimal functionality in the body, the RBC shape is very important, and a variation in shape may lead to drastic changes in oxygen availability in the body. An extreme example for this is sickle cell anemia, which is triggered by polymerization of hemoglobin eventually leading to prominent cell shape changes and local clogging of the blood circulation^19^.

Experimental manipulation of RBC shape changes can be achieved by reducing or increasing the area-to-volume ratio which leads to other geometries than the typical discocyte and changes of the mechanical properties in general. To manipulate the geometry of cells, two obvious approaches are reasonable. In order to manipulate the RBC surface area they can be treated with specific amphipathic agents^20,21^. These agents can either intercalate into the outer or inner hemileaflet of the RBC phospholipid bilayer and cause a disturbance resulting in the formation of echinocytes or stomatocytes, respectively^22,23^. In these cases the area relation of the lipid bilayer is altered which leads to a morphological change. Another trigger to change the shape of RBCs is to change the tonicity (effective osmotic pressure gradient) of the surrounding media^24,25^. In this way the cell volume can be changed via osmosis and as a result from this change in the area-to-volume the cell adopts a certain shape. The two methods outlined above are reversible in a way that either the tonicity of the medium can be readjusted or the RBCs can be washed free of the amphipathic agents. However, these operations are rather slow (osmosis takes several tens of seconds) and changing the surrounding media is more complicated when direct observation of the cells is desired^26^.

Here we demonstrate modulation of the membrane area of RBCs and other eukaryotic cells such as myoblasts and cancer cells in a fast and reversible way via photoirradiation. Using a molecular photoswitch as a photoresponsive membrane intercalating agent allows optical manipulation of the membrane area. Light as an external stimulus provides a unique spatiotemporal resolution that can be used to control the shape of these photoresponsive living cells. We note that the integration of a molecular photoswitch into various soft materials is a versatile strategy to achieve photoresponsive systems^27–29^. From the numerous molecular photoswitches, azobenzene is one of the best characterized^30–32^. It photoisomerizes from the thermodynamically stable *E*-isomer to the metastable *Z*-isomer by exposure to UV light (λ ≈ 365 nm). The back-isomerization is either triggered by visible (vis) light (λ ≈ 460 nm) or by a comparatively slow thermal process that takes around 2 days^33,34^. The photoisomerization in contrast is very fast and completed in picoseconds^35^. These photoisomerization processes are highly reversible and irradiation results in an equilibrium state, the photostationary state (PSS), that depends on the wavelength used. The isomerization induces a change in the molecular geometry and in the dipole moment of the azobenzene (from 0 D in the *E*-isomer to 3.0 D in the *Z*-isomer)^36,37^. Because of these photophysical properties, their straightforward synthesis and simple modification, azobenzenes are nowadays widely used in biological applications and are even being tested for their anti-cancer activity^38–41^. By introduction of azobenzenes into nucleic acids, peptides or even cellular receptors and trans-membrane channels, versatile photocontrolled systems could be generated^42–48^. Photopharmacology is an emerging field that combines the research that employs photoswitches for remote optical control of active compounds^49–51^. Modifying lipids with azobenzene moieties yields photomodulated membranes and liposomes with switchable permeabilities^52–55^. Studies on vesicles as model membranes for living cells showed that with photoswitches attached to the membrane, the shape or the membrane capacitance can be reversibly photocontrolled^15,56–62^. Furthermore, a membrane targeted photoswitch can be employed to modulate neuronal firing^63^. Only recently was it possible to use an azobenzene derivative as a viscosity probe for cell membranes^64^. However, to the best of our knowledge there are no studies that introduce photoswitches into RBCs or any other cell types in order to manipulate the membrane area of living cells in situ and reversibly. Therefore, the presented work is the combination of a very simple membrane intercalating molecular photoswitch (Azo-SO_3_H) that enables a fast and reversible surface area change of living cells via photoisomerization.

## Results and discussion

**Fig. 1:**
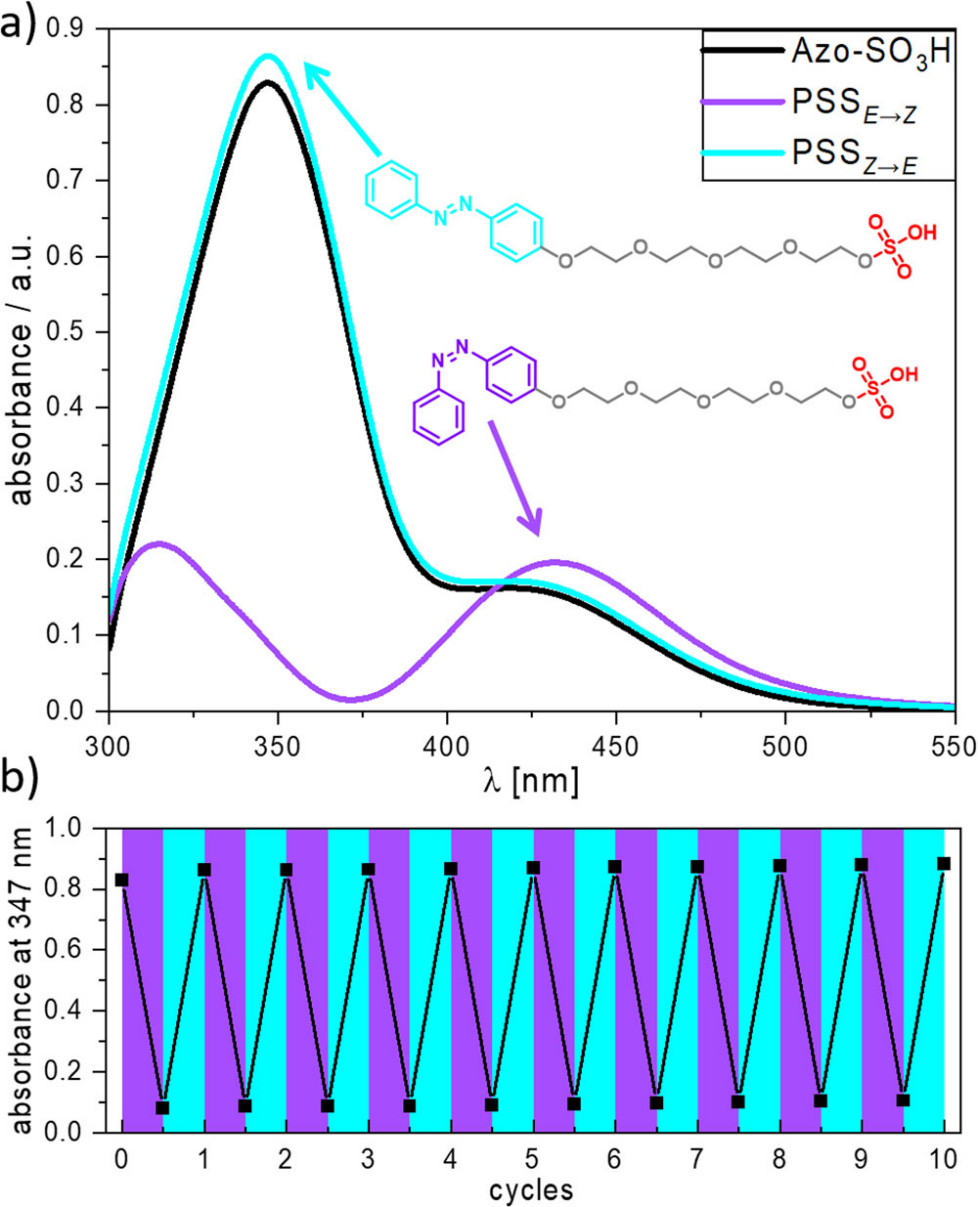
Reversible photoisomerization of Azo-SO_3_H. **a** Molecular structure of the *E*- and *Z*-isomer of photoswitch Azo-SO_3_H and UV/vis absorption spectra in the initial state and the two photostationary states PSS_E→Z_ after 5 s UV (365 nm) irradiation and PSS_Z→E_ after 10 s blue (465 nm) irradiation (80 µM in DMEM). **b** Photoswitching cycles (*n* = 10) for Azo-SO_3_H using alternating UV (5 s at 356 nm) and blue irradiation (10 s at 465 nm).

As a photoresponsive membrane intercalating agent, an amphiphilic anionic azobenzene derivative was synthesized and analyzed upon its photophysical properties. In Fig. 1a the molecular structure of Azo-SO_3_H is shown together with the UV/vis absorption spectra of Azo-SO_3_H in basal medium (DMEM) for the initial state and both photostationary states (PSS), PSS_*E*→*Z*_ for the *Z*-isomer and PSS_*Z*→*E*_ for the *E*-isomer after irradiation with UV and vis light, respectively. Azo-SO_3_H shows the typical UV/vis absorption bands for azobenzenes with a strong peak around 347 nm caused by the π→π* transition and a shoulder at around 420 nm attributed to the *n* → π* transition for the *E*-isomer. The *Z*-isomer shows a drastic decrease in absorbance around the π→π*-band and an increase in the region of the *n* → π*-band leading to a red-shifted peak around 431 nm. To show the efficient photoisomerization process, multiple switching cycles were measured. In Fig. 1b 10 switching cycles are shown. Using a UV LED (365 nm) the PSS_*E*→*Z*_ can be reached in less than 5 s while irradiation for 10 s with a blue LED (465 nm) yielded the PSS_*Z*→*E*_. The completion of the photoisomerization was measured at the peak of the π→π*-band absorbance at 347 nm. Measured over 10 cycles, Azo-SO_3_H showed no fatigue and demonstrates an excellent photoisomerization behavior. In addition, the thermal half-life time of the *Z*-isomer in aqueous solution was measured (see Supplementary Fig. 1–3). It was found to be around 64 h and is consistent with the data for other azobenzenes^33,34^.

The amphiphilic nature of Azo-SO_3_H was confirmed by surface tension measurents indicating a critical micelle concentration of 1.2 mM for *E*-Azo-SO_3_H and a much lower surface activity for *Z*-Azo-SO_3_H throughout the tested concentration range (c = 0.1–5.0 mM, see Supplementary Fig. 4). In order to gain a better understanding of the behavior of Azo-SO_3_H in the presence of a phospholipid bilayer the partition coefficient (*n*-octanol/DMEM) was determined via the shake flask methode^65^. The interaction of solutes towards cell membranes can be characterized by the membrane partition coefficient which represents the solute distribution between the aqueous phase and the membrane^66–69^. The smaller the mean log(*P*_OW_), the more hydophilic is the solute. Instead of using the standard *n*-octanol/H_2_O system a basal medium (DMEM) was choosen as the aqueous phase to better simulate in vitro conditions. The *E*-isomer has a mean log(*P*_OW_) = 0.88 ± 0.21 whereas the *Z*-isomer shows a lower mean log(*P*_OW_) = 0.51 ± 0.16 (see Supplementary Fig. 5). This demonstrates the large influence of the photoisomerization on the hydrophobicity of Azo-SO_3_H. In general, both isomers are more soluble in the hydrophilic phase than in the hydrophobic phase, indicated by a log(*P*_OW_) < 1. But with a ~40% lower log(*P*_OW_) value, the *Z*-isomer is more likely to be dissolved in the aqueous phase than the *E*-isomer.

To test the hypothesis that indeed the *E*- and *Z*-isomers interact in different ways with the membrane of RBCs we tested whether cells can trap, and hence partially remove, Azo-SO_3_H from the solution. If the *E*-isomer intercalates strongly into a phospholipid bilayer and the *Z*-isomer does not or less, the fraction of Azo-SO_3_H bound to the RBC membrane should be different. A constant amount of RBC containing medium (1 µL) was added to a solution of either *E*- or *Z*-Azo-SO_3_H (1 mL, *c* = 147 µM, irradiated with vis light to obtain *E*-Azo-SO_3_H or with UV light to obtain *Z*-Azo-SO_3_H) in HEPES (*c* = 300 mM, pH 7.0) to yield an approximate RBC concentration of ~10^7 ^mL^−1^. In this way both isomers can interact individually with the membrane. Upon removal of the RBCs via centrifugation and filtration the absorbed Azo-SO_3_H is also removed, leaving only the Azo-SO_3_H that did not bind to the plasma membrane in solution. Measuring the concentration of photoswitch in the supernatant via absorption spectroscopy after irradiation with vis light to yield the initial PSS gives the difference to the initial concentration (before addition of RBCs). For the *E*-isomer a mean loss of 62 ± 2.0% was measured, meaning that only 38% of the Azo-SO_3_H remained in the aqueous phase. For the *Z*-isomer a mean loss of only 35 ± 2.8% was found (see Supplementary Fig. 5-7). In contrast to the *E*-isomer the majority of Azo-SO_3_H (65%) remained in the aqueous phase. These experiments show that the *Z*-isomer has a ~40% lower affinity to bind to the plasma membrane of RBCs. This is consistent with the log(*P*_OW_) values determined for Azo-SO_3_H and shows that both methods can very well explain the different affinity of the two isomers towards a phospholipid bilayer. These experiments give strong evidence that indeed the more hydrophobic *E*-isomer has a higher tendency to intercalate into the plasma membrane of RBCs than the more hydrophilic *Z*-isomer.

**Fig. 2:**
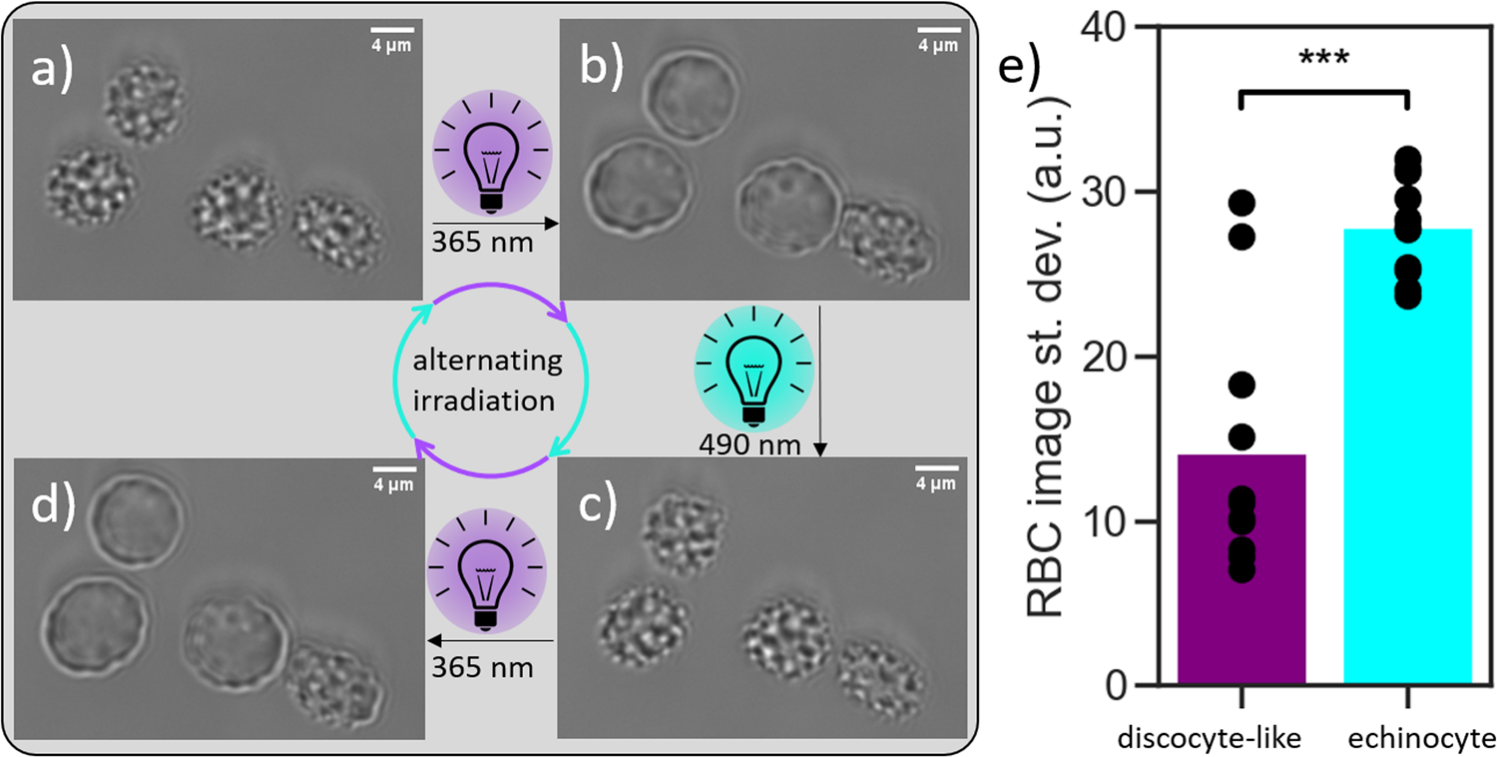
Microscopy images of free-floating RBCs. **a**–**d** The morphology changes depend on the reversible photoisomerization of the Azo-SO_3_H from E-Isomer (echinocyte, (**a**) and (**c**)) to Z-Isomer (discocyte-like, (**b**) and (**d**)). **e** Quantification of the inhomogeneity of *N* = 3, *n* = 13 RBCs via the standard deviation of the pixel values. The difference between the discocyte-like and echinocyte is significant (two-sided *t* test, *p* = 7 × 10^−7^).

To visualize the effect of Azo-SO_3_H on the RBC membrane and the overall morphology of the cell, microscopic studies were employed on free-floating RBCs. In a typical experiment, RBCs were carefully added to a solution of Azo-SO_3_H in DMEM (*c* = 1.0 mM, i.e. below the critical micelle concentration). Monitoring of the RBC shape was started upon sedimentation. Initially, nearly all cells appear in the crenated cell shape (echinocytes, see Fig. 2a), identified by spicules evenly distributed around the cell. We assume that this is because the photoswitch is in its thermodynamic resting state (*E*-isomer) and immediately intercalates into the plasma membrane of the RBCs and hence increases the membrane area. Observation for several hours did not detect any change of the morphology of these cells. However, upon irradiation with UV light (365 nm) a very fast and pronounced shape change is observed. From the echinocyte form, the RBCs transform into a irregularely contoured discocyte-like shape in less than 2 s (see Fig. 2b, c). Our measurements suggest that this shape change originates from the geometry and dipole moment change in the molecular photoswitch. The *E*-isomer of Azo-SO_3_H intercalates into the outer hemileaflet of the RBC plasma membrane which leads to an increased membrane area and therefore to a changed surface area-to-volume ratio of the RBC (echinocyte). Once the molecular switch is photoisomerized into the *Z*-isomer, significantly less (~40%) Azo-SO_3_H remains bound to the lipid bilayer and therefore the surface area-to-volume ratio changes, leading to a discocyte-like shape (see Supplementary Fig. 10). The term discocyte-like also includes slightly oblate cells. By photoisomerization back into the *E*-isomer (490 nm), Azo-SO_3_H rebinds to the surface of the RBCs and thus the echinocyte can be observed again (Fig. 2c). This completes an isomerization cycle of Azo-SO_3_H and with it a reversible morphology transformation from the discocyte-like to echinocyte and back. The process is very fast, highly reversible and can be performed multiple times without fatigue or lysis of the cells (see Supplementary Movie 1). To quantify the visible change, we calculated the standard deviation of the pixel value of images of free-floating RBCs in discocyte-like and echinocyte form as a measure of the homogeneity of the structure of the RBC image. A significant difference between the two stages is observed (see Fig. 2e). It has to be mentioned here that not all RBCs show an equal response (e.g. Fig. 2a–d lower right). This is expected, as the shape changes of RBCs are known to occur at defined surface-to-volume ratio limits, but due to aging, the area and volume of a RBC changes over its 90 day lifetime^70,71^. To test if indeed the surface-to-volume differences between the cells are the reason for the differences in cell response, we systematically increased the volume by adding pure water to the medium which results in hypoosmotic conditions. Indeed, we find that cells that initially stayed in the echinocyte state did now undergo a shape change upon UV illumination (see Supplementary Fig. 11). Hence, the relative area increase induced by the Azo-SO_3_H may cause a different extent of shape change as function of RBC age.

**Fig. 3:**
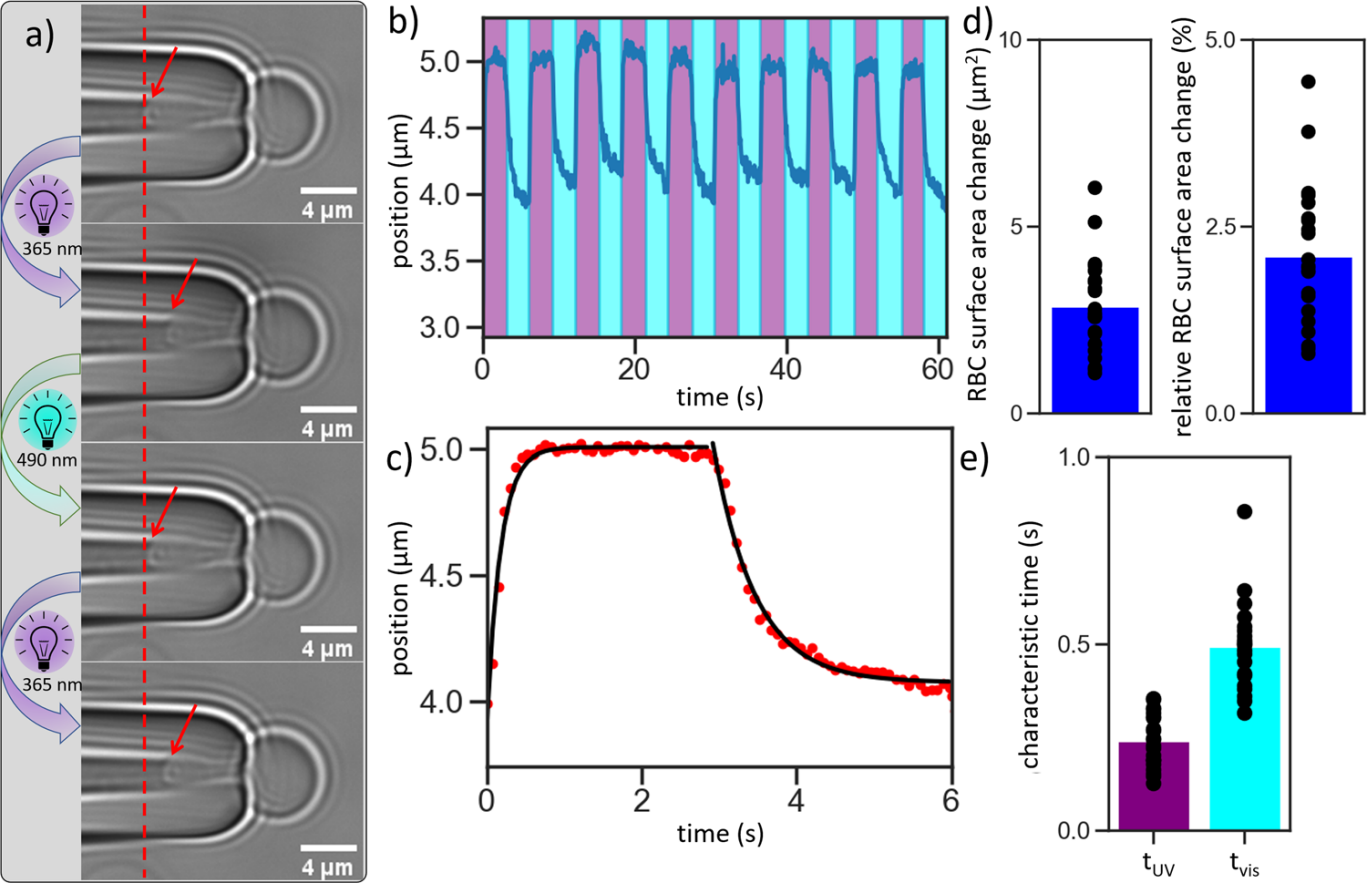
Analysis of aspirated RBCs with low applied tension of ca. 10^-5 ^N/m. **a** Microscopy images from an aspirated RBC during photoisomerization with consequent movement of the membrane (red line and arrow as a guide). **b** Tracking of the movement of the membrane inside the micropipette while switching for 10 cycles. **c** Time-dependent movement of 10 cycle shifted on top of each other, averaged and fitted (black line). **d** Analysis of the absolute change in membrane area and in relation to the overall surface. Data show 22 experiments on 6 different days. **e** Characteristic switching times, *t*_UV_: UV light switch, *t*_vis_: vis light switch. Data show *N* = 6, *n* = 22 experiments.

To further quantify the effect of the photoswitch on the RBC mechanics, we perform micropipette aspiration on the RBCs. Micropipette aspiration is a versatile tool to study the biomechanics of living cells and has been used extensively on both soft and rigid cells^11,12,72–75^. Connecting a small glass pipette with an inner diameter of 1–2 µm to a precisely height-tunable water reservoir allows to build up a controlled suction pressure that can be used to partially partially aspirate cells (see Supplementary Fig. 12). Analyzing the movement of the part of the cell membrane that is sucked into the micropipette allows to measure small differences of the membrane area with a temporal resolution of the camera frame rate (75 ms). In Fig. 3a, partially aspirated RBCs are depicted. The biggest part of the cell is outside of the pipette and in spherical shape (see Supplementary Fig. 13), while a small part of it is sucked into the pipette. The part inside the pipette adjusts to the dimension of the pipette and is capped by a hemispherical end. The hemispherical end of the cell (tongue) is highlighted throughout Fig. 3a with red arrows. In this picture compilation, the red dotted line indicates the position of the tongue in the initial state that is shown in the top panel of Fig. 3a. Here the *E*-isomer of Azo-SO_3_H is intercalated into the RBCs plasma membrane and increases the membrane area of the outer hemileaflet. Without changing the height of the water reservoir this state is stable over several minutes. Upon irradiation with UV light the tongue immediately retracts a little out of the pipette, as can be seen in the second panel of Fig. 3a. This clearly shows the difference in the tongue position inside the pipette before and after photoirradiation. Upon UV irradiation, the *E*-isomer of Azo-SO_3_H tends to leave the bilayer and in comparison to the initial state, the surface area is decreased. This decrease leads to a remodeling of the membrane structure and retraction from the pipette. In accordance with the photoisomerization of Azo-SO_3_H in solution (Fig. 1), the membrane area increase of the RBC is fully reversible for at least 10 cycles. A closer look at the part of the cell that is outside the pipette shows how the RBC first remodels the membrane, shown by strong fluctuation and then gives in to the suction pressure and extends the membrane further into the pipette (see Supplementary Movie 2). This can be attributed to the back-isomerization of the photoswitch to the *E*-isomer and a re-intercalation of Azo-SO_3_H into the membrane. We note that upon addition of Azo-SO_3_H, the RBC immediately change from a discocyte-like shape to an erythrocyte shape (see Supplementary Movie 3) and revert to a round shape upon aspiration to the micropipette (see Supplementary Movie 4). In Fig. 3b the tracked position of the tongue inside the pipette against the time is depicted for such an experiment. This clearly shows the fast and reversible movement of the cell membrane in and out of the micropipette in response to the photoisomerization of Azo-SO_3_H. This switching cycle can be repeated many times without fatigue or damage of the cell. In a typical experiment 10 of these cycles were performed on individual RBCs.

A closer inspection of the time that is needed for the membrane to reach its final position in the micropipette (relaxation time) reveals two different behaviors. In Fig. 3c the time-dependent movement of 10 cycles are synchronized, averaged and plotted as red dots. The black curve shows two exponential fits of the data, for the illumination with UV and vis light, respectively. Starting here in the resting state of the system (*E*-isomer of Azo-SO_3_H) the tongue immediately shows a large and fast movement out of the pipette after irradiation with UV light (*t*_UV_). This switch takes only *t*_UV_ = 240 ± 70 ms to reach a plateau, where it stays until irradiation with vis-light. In contrast to this short relaxation time the switch with vis light is over a factor two slower, being *t*_vis_ = 490 ± 110 ms (Fig. 3e). This indicates that two different processes are at work. It is most likely that the photoisomerization itself is not the rate determining step as it is usually very fast (~10^−12 ^s)^76,77^. Therefore either the membrane extension vs. membrane contraction happen on different timescales or the detachment of Azo-SO_3_H from the plasma membrane is faster than the intercalation. Indeed, when irradiated with vis light, it can be seen that the membrane first shows stronger fluctuations and after that extends into the pipette. In contrast, when irradiated with UV light no increase in membrane fluctuations is visible and the first and only observable response is the retraction of the membrane out of the pipette (see Supplementary Movie 2). Also shown in Fig. 3 are the summarized results of a statistical analysis of the micropipette aspiration experiments. Here the average RBC surface area change is shown in Fig. 3d. An average absolute area change of 2.9 ± 1.2 µm^2^ was found, which corresponds to a relative surface area change of 2.1 ± 0.9%. As described above, we propose that this change in area is due to *E-*Azo-SO_3_H entering and Z-Azo-SO_3_H leaving the membrane. Assuming that the Azo-SO_3_H surfactant has roughly half the footprint of a lipid, a 2% area change reflects a 4 mol% Azo-SO_3_H content of the membrane.

**Fig. 4:**
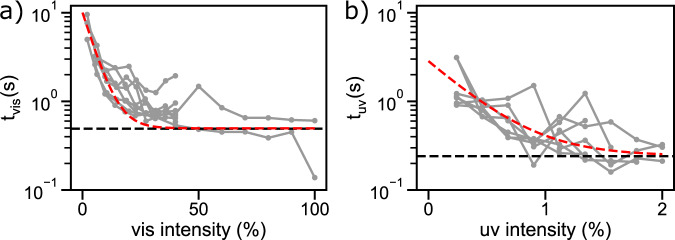
Relaxation times of aspirated RBCs depends on the light intensity. **a** Relaxation after vis light irradiation. **b** Relaxation after UV light irradiation. The black dashed line is the average relaxation time after irradiation with 100% vis or 2% UV intensity, respectively (see Fig. 3e). For light intensity translation from % to µW see Supplementary Fig. 8. The red dashed line is an exponential function of the fitted characteristic switching times with increasing irradiation intensity and serves as a guide to the eye, while the gray curves represent individual measurements. Data show *N* = 3, *n* = 7 (**a**) and *N* = 3, *n* = 8 (**b**) experiments.

To investigate whether the rate of the photoisomerization can change the relaxation times, micropipette experiments were performed where the intensity of the vis light irradiation was gradually increased over multiple cycles while the UV irradiation was kept constant to always return to the PSS_*E→Z*_. Furthermore, inverse experiments with increasing UV light intensities and constant vis light intensity were performed. In Fig. 4 the relaxation times of the vis and UV irradiation are plotted against the light intensity. It can be seen that for low vis intensities *t*_vis_ is higher and decreases with increasing vis intensities. Also, at a certain vis intensity, the relaxation time reaches a plateau value for *t*_vis_ that is similar to the cyclic experiments with constant vis intensity (Fig. 4a). The same trend can be observed for increasing UV intensities (Fig. 4b), albeit saturation occurs already at lower light intensities. This shows that at low irradiation intensities, complete photoisomerization is slowed down and thus the relaxation times are high. At a certain intensity, however, the relaxation times are no longer accelerated by the faster photoisomerization and other effects limit the relaxation rate. The difference in the rate between the two times *t*_UV_ and *t*_vis_ at high light intensity could be attributed to the asymmetry between diffusion of the Azo-SO_3_H from the surrounding water into the membrane, and diffusion out of the membrane, although more research is needed to confirm this.

Additionally, analysis on free-floating RBCs also showed a dependency of the relaxation times on the irradiation intensity, with again the vis switch being slower than the UV switch (see Supplementary Fig. 9). However, as the analysis of the free-floating RBCs is less accurate, only a qualitatively agreement between the observations on these RBCs can be stated. Thus, the differences in relaxation times are not due to the difference between membrane extension into the pipette or withdrawing from it, but clearly result from the intercalation or detachment of Azo-SO_3_H from the plasma membrane.

We note that as the RBC is retracting out of the micropipette under a constant suction pressure, the cell actually performs mechanical work. The suction pressure can be easily calculated by the height difference of the water reservoir. Combined with the diameter of the micropipette and the length of the tongue movement the performed work can be calculated. In a typical switching experiment the RBCs performed work in the order of *W* = 6.7 ± 2.4 × 10^3^ *k*_B_*T*.

**Fig. 5:**
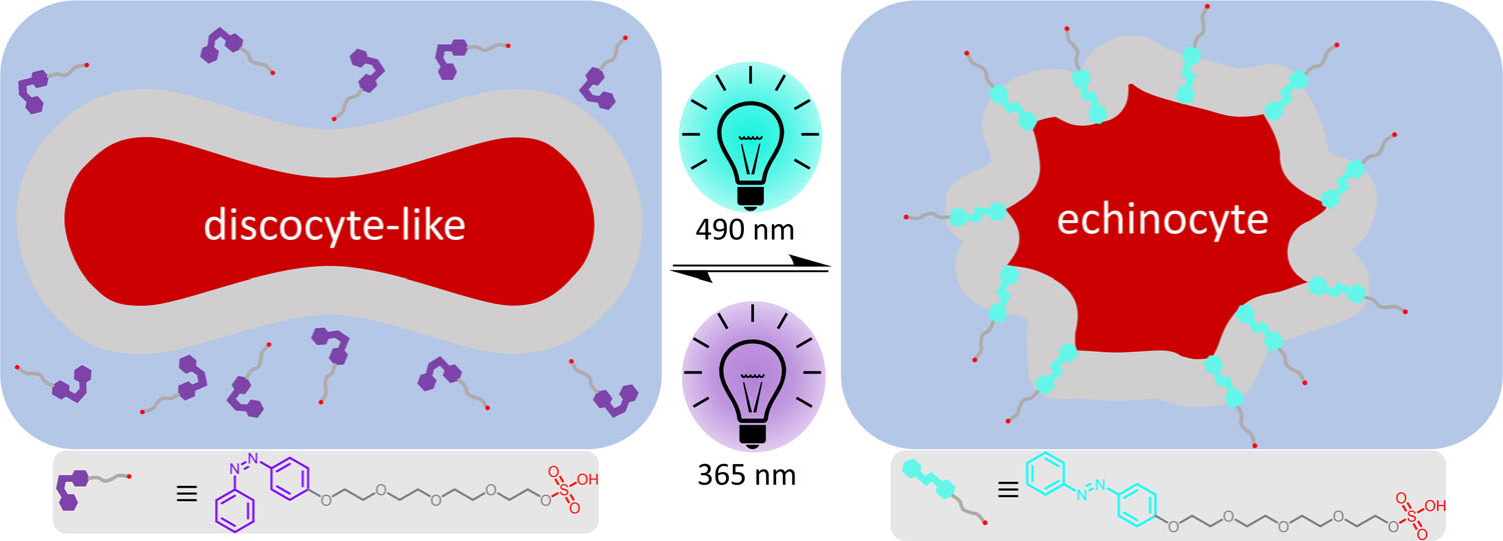
Photoresponsive shape transformation of RBCs. Schematic representation of the photoresponsive, reversible incorporation of Azo-SO_3_H into the RBC plasma membrane and the corresponding switch between the discocyte-like and echinocyte morphology.

Based on the presented results, the proposed mechanism of photoinduced membrane effects on RBCs is shown in Fig. 5. Depending on the photoisomerization of the azobenzene (Azo-SO_3_H) it is either mostly freely dissolved in the medium (*Z*-isomer, left side) or intercalated into the plasma membrane of the RBC (*E*-isomer, right side). The photoisomerization can be triggered fast and reversibly by UV light (365 nm, *E*-to-*Z*) or vis light (490 nm, *Z*-to-*E*). The photoisomerization and subsequent intercalation into the membrane or diffusion out of the membrane causes a fast and strong response by RBCs. The more hydrophilic *Z*-isomer of Azo-SO_3_H has little affinity for the membrane and the RBCs are in a discocyte-like shape, while the more hydrophobic *E*-isomer of Azo-SO_3_H intercalates into the membrane and the RBCs are in a crenated echinocyte shape. The two morphologies can be converted into one another upon irradiation with light in a highly reversible and fast manner.

In a recent study on photoinduced morphological changes in giant unilamellar vesicles (GUVs) Georgiev et al. showed that an *ortho*-tetrafluoro-azobenzene could be used to reversibly change the membrane area of DOPC GUVs^15^. In that case, both isomers intercalate into the phospholipid bilayer but upon photoisomerization into the *Z*-isomer the membrane area is increased. Using MD calculations the authors concluded that the different orientation of the photoswitch inside the membrane is responsible for the observed membrane area change. In the present work the opposite behavior of the photoswitch is observed: the *E*-isomer intercalates into the membrane whereas the *Z*-isomer prefers the aqueous phase. The different behavior of the azobenzenes could be due to different dipole moment changes for the two photoswitches. Azobenzene has a dipole moment change of about 3.0 D going from the *E*-isomer (0.0 D) to the *Z*-isomer (3.0 D)^37^. Although the dipole moment change of azobenzene increases with fluorine substituents, additional substituents on the *para*-position can drastically reduce the dipole moment change to merely 0.2 D^78^. It is therefore possible that the *ortho*-tetrafluoro-azobenzene has a very small dipole moment change whereas the Azo-SO_3_H retains the large dipole moment change from the parent azobenzene. Another important factor is the increased partition coefficient of fluorinated compounds^79^. Fluorine substituents strongly increase the hydrophobicity of the photoswitch so that it prefers to stay in the hydrophobic membrane rather than in the hydrophilic water phase irrespective of the photoisomerization. Thus, a less hydrophobic photoswitch may lead to a very different membrane interaction.

**Fig. 6:**
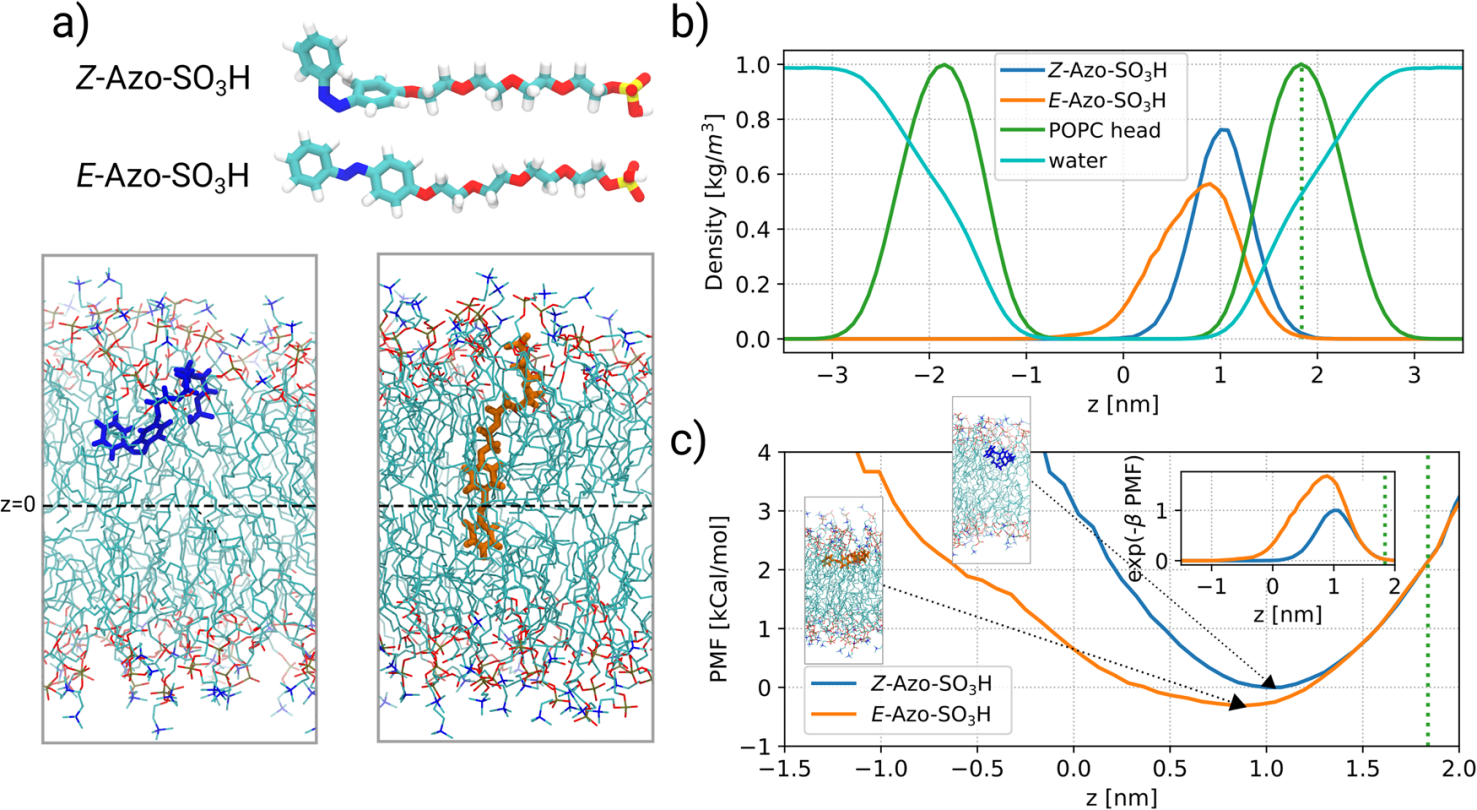
Molecular dynamics simulation of Azo-SO_3_H a lipid membrane. **a** Molecular structure of the Azo-SO_3_H isomers and snapshots of the *E*-isomer and Z-isomer inside the POPC membrane. The approximate position of the center of the membrane (z = 0) is shown by a dashed line. **b** Number densities of Azo-SO_3_H as well as the POPC head group and the water molecules along the membrane normal (z-axis). The number densities for the POPC head group and water molecules were rescaled and multiplied by 0.017 and 0.01, respectively, so that all the profiles can be conveniently shown in one plot. The average value of the density profile of the POPC head group is shown by a dotted line. **c** The PMF profiles of the Azo-SO_3_H molecules along the membrane normal are shown. The snapshot of the two isomers for the minimum of the free energy is represented. The PMF-profiles are shifted so that they agree at the average position of the POPC head group. The resulting distribution exp(-β PMF) for the two isomers is shown in the inset.

The proposed mechanism of photoreversible membrane intercalation is supported by molecular dynamics (MD) simulations. At the start of the simulations, the *E*- and *Z*-isomers of Azo-SO_3_H (Fig. 6a) are located in the solution while in the course of the simulations they are absorbed by the membrane. Due to the high affinity, they remain in the membrane for the rest of the simulation (Fig. 6a). Interestingly, the molecules are far more stretched in the membrane than in the solution (Fig. 6a). Furthermore, the azobenzene unit of the molecules always remains in the same leaflet and points towards the center of the membrane (Fig. 6a, b). Further MD simulations indicate that the transition to the other leaflet is suppressed by the hydrophilic ethylene glycol chain (see Supplementary Fig. 21).

The density profiles of Azo-SO_3_H (Fig. 6b), determined from the position of the center of mass of the azobenzene unit, show that the *E*-isomer traverses mainly the hydrophobic core of the membrane, near the center of the membrane, whereas the *Z*-isomer approaches the outer side of the membrane. Closer information can be gained from the potential of mean force (PMF) (Fig. 6c), which can be calculated from the density profiles (Fig. 6b), using the inverse Boltzmann formula PMF = −k_B_T ln ρ(z), where k_B_ is the Boltzmann factor and T is the temperature. Interestingly, when approaching the center of the membrane, there is only a small increase of the free energy for the *E*-isomer as compared to the *Z*-isomer.

As reported by Georgiev et al. for *ortho*-tetrafluoro-azobenzene^15^, the increase of the free energy upon leaving the membrane does not depend on the isomer. Indeed, also in our case we can overlap the PMF profiles in the range captured by our simulations. We note in passing that even the increase in free energy with the change in position is very close to the data of Georgiev et al.^15^ ( < 25% difference). Starting from these appropriately shifted PMF profiles, we can estimate the relative contribution of the inner-membrane behavior of the two isomers to the partitioning. For this purpose we calculated the distribution exp(-β PMF) (Fig. 6c,inset), where β is equal to 1/(*k*_B_*T*). The integral over this distribution for the *Z*-isomer is around 0.43 times the distribution for the *E*-isomer. We note that this reduction is close to the overall reduction of the partitioning coefficient of 0.67 = 1exp(−0.4)), seen in our experiments from the comparison of both isomers (see Supplementary Fig. 5). Thus, we can conclude that the higher affinity of the *E*-isomer to the hydrophilic region of the membrane has a significant impact on the resulting partitioning. Interestingly, for the fluorinated azobenzene no significant difference for the isomers was observed in the hydrophobic region of the membrane^15^. These observations support the general arguments mentioned above.

**Fig. 7:**
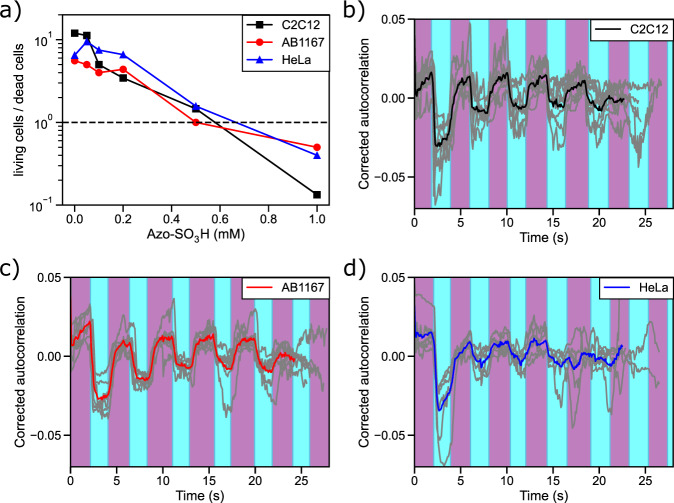
Photomanipulation of myoblasts and cancer cells with Azo-SO_3_H. Viability assay of the three different cell lines (**a**) and the corrected autocorrelation profiles that quantify the changes in cell morphology over 5 cycles at concentrations of 0.5 mM Azo-SO_3_H (**b**–**d**) on C2C12 (*N* = 3, *n* = 8, **b**), AB1167 (*N* = 3, *n* = 7, **c**) and HeLa cells (*N* = 3, *n* = 6, **d**).

In order to demonstrate the broader scope of this photoresponsive membrane intercalating agent beyond RBCs, three different cell lines were investigated: Murine myoblasts (C2C12), human myoblasts (AB1167) and cervical cancer cells (HeLa). In a first step the cell viability vs. increasing amounts of Azo-SO_3_H were studied via a viability assay (see Fig. 7a and Supplementary Figs. 15–18). Up to 0.2 mM, the photoswitch had very little effect on the viability of all cells. Even at a concentration of up to 0.5 mM the critical limit of 50% survival after 24 h was not reached (ratio living/dead cells >1). Therefore, further experiments with these living cells were performed at concentrations of 0.5 mM. Interestingly, all three cell lines showed qualitatively similar membrane movement to that of RBCs, meaning that upon UV irradiation the cells contracted while under vis light they expanded again (for controls see Supplemental Fig. 14). In contrast to the RBCs, these cells were investigated while being adherent to the glass substrate, so that only those parts of the cells that were not adherent showed significant membrane movement (see Supplementary Movies 5–7). To quantify the reversible photoresponsive cell membrane movement an autocorrelation of the cell image sequences was calculated over the time of five switching cycles. In Fig. 7b–d the corrected autocorrelation is shown for all three cell lines. All cells show a significant movement upon illumination change as the autocorrelations show clear peaks. Upon UV illumination the autocorrelation drops and stabilizes after a short time until it increases under vis light again to a plateau. One such switching cycle can be repeated at least four times, before the autocorrelation completely flattens due to (intra)cellular drift. Depending on the cell type and the Azo-SO_3_H concentration the intensity of the membrane movement decreases with each cycle and is eventually no longer detected. In the absence of Azo-SO_3_H or without alternating UV/vis illumination the cells do not show any change in the corrected autocorrelation (see Supplementary Fig. 14). This experiment impressively shows that the photoresponsive membrane intercalating agent Azo-SO_3_H not only is applicable to RBCs but also to other living cells. Furthermore, a very similar membrane contraction and expansion was observed, which suggests that the same interactions between the photoswitch and the membrane of these cells exists as observed for RBCs.

To summarize, in this work an amphiphilic azobenzene molecular photoswitch (Azo-SO_3_H) was synthesized. In basal medium, it showed the typical photoisomerization of an azobenzene. The partition coefficient (*n*-octanol/DMEM) showed that the *Z*-isomer has a ~40% lower probability to be in the hydrophobic phase resembling the phospholipid bilayer of cells. In vitro studies on RBCs, as living model systems, lead to the result that indeed the *Z*-isomer is ~40% less incorporated into the plasma membrane. Microscopical studies of RBCs elucidate these findings and show a photoresponsive and reversible shape change from a discocyte-like to an echinocyte, and back. Micropipette aspiration experiments gave further insight into the dynamics of the photoinduced RBC shape change. A reversible relative surface area change of 2.1% was observed during photoirradiation cycles. The photoresponsive RBCs were found to be able to perform work against a suction pressure in the order of *W* = 6700 *k*_B_*T* under the used experimental conditions. Furthermore, a similar membrane expansion and contraction was observed for three different cell lines indicating that indeed Azo-SO_3_H can be used to photomodulate a variety of living cells. Our experiments confirm the hypothesis that the *E*-isomer of Azo-SO_3_H intercalates into the plasma membrane and thus increases the total membrane surface area while the *Z*-isomer is excluded from the membrane leading to a reduced surface area. In short, a highly accessible and straightforward protocol is presented for the fast and reversible manipulation of the surface area of living cells by simple photoirradiation. This tool will therefore open the door for new experiments to test the importance of membrane area for fundamental cell biological processes. Since light can be applied highly localized and precisely timed, we are now in the position to manipulate membrane area and hence membrane tension precisely. Given the recent insights that membrane tension is a key parameter for cell differentiation^5,6^, cell migration^7–9^ and cell volume regulation^10^, we hope that this protocol can be used regularly in biophysical studies and lead to new insights into the behavior of biological membranes.

## Methods

### Preparation of RBCs

Human red blood cells were freshly prepared before each experiment by finger pricking of a healthy and voluntary donor. 20 µL of blood were diluted in 250 µL of MEM (Minimum Essential Medium, Gibco) containing 10% fetal calf serum (FCS, Sigma-Aldrich). RBCs were washed twice in the described buffer by centrifugation (5 min, 100 g) and aspiration of the supernatant. The sedimented RBCs after centrifugation are referred to if RBCs were used. The Ethics Committee of the University of Göttingen approved this study (01112021).

### Fluorescent labeling of RBCs

ATTO 488 NHS ester (Sigma-Aldrich), dissolved in PBS, is added to the RBCs to a final concentration of 0.15 mM and incubated at room temperature under continuous stirring. After 30 min, the RBCs were washed three times in the described buffer by centrifugation to remove excess dye.

### Cell culture

All cell lines were cultivated in uncoated glass bottom dishes (CELLview 35/10 mm, Greiner Bio-one) at 37 °C and 5% CO_2_ in a humidified incubator. Murine C2C12 myoblasts (ATCC) and human HeLa cervical cancer cells (ATCC) were cultured in high glucose Dulbecco’s Modified Eagle Medium (DMEM, Capricorn) containing 10% fetal calf serum (FCS, Sigma-Aldrich) and 1% penicillin-streptomycin (Gibco). For human AB1167 myoblast cells (provided bv Vincent Mouly, Institute of Myology, Paris, France) the Skeletal Muscle Cell Growth Medium kit (Promocell) supplemented with 15% FCS and 1% penicillin-streptomycin was used. The cells were thawed in 10 ml of the respective media, centrifuged for 4 min at 400 x g and seeded in the dishes one day prior to the experiments and Azo-SO_3_H addition. Videos of photomanipulation of cells were recorded directly after Azo-SO_3_H addition. The measurements were performed in three independent biological replicates for each cell line with the number of measurements as indicated in the figure legend.

### Cell viability

HeLa, C2C12 and AB1167 cells were seeded into 6-well plates and cultured under the conditions described above. The next day, Azo-SO_3_H (*c* = 10 mM in H_2_O) was added to the culture to yield a total Azo-SO_3_H concentration of 0.05 mM, 0.1 mM, 0.2 mM, 0.5 mM and 1 mM, respectively. As a negative control, 200 µl of H_2_O was utilized (amount corresponds to 1 mM Azo-SO_3_H solution used). After 24 h, the cell culture media was aspirated and kept for dead cell quantification. The adherent cells were washed with PBS^-/-^ (Sigma) once and removed from the dishes using Trypsin/EDTA solution (0.05%/0.02%, Sigma). Live and dead cells were counted for each condition using the LUNA-II automated cell counter (Logos Biosystems) following a Trypan Blue staining (0.4%, Sigma-Aldrich).

### Determination of partition coefficient *n*-octanol/DMEM

The partition coefficient of Azo-SO_3_H between *n*-octanol and DMEM was determined according to an OECD Guideline (Test No. 117, Shake Flask Method) at r.t^65^. DMEM was used here instead of pure water because this is the medium the experiments on RBCs were done in and are therefore more relevant for the explanation of the found experimental results. Prior to use, *n*-octanol and DMEM were mutually saturated. Stock solutions of Azo-SO_3_H (200 µM) in *n*-octanol and DMEM (pH 8.1) were prepared. 12 Experiments were conducted with compositions according to Supplementary Table S1. The solutions were filled in centrifugation tubes (CELLSTAR® TUBES, 15 mL) and shaken for 5 min by hand. No. 1–6) were irradiated before and while shaking with vis light (465 nm, 5 min) and 7-12) were irradiated with UV light (365 nm, 5 min) before shaking and briefly before centrifugation, also these samples were handled in the dark (red lights) from the first UV irradiation on. After mixing, the tubes were centrifuged (5 min, 1000*g*) to separate the phases and consequently 1 mL of both phases were carefully removed and analyzed. The analysis was done via UV/vis spectroscopy. For this a calibration of Azo-SO_3_H in *n*-octanol and DMEM was used to determine the concentration in both phases (see Supplementary Figure S5). Important to note is that the samples 7–12) were irradiated with vis light after taking the samples to obtain the *E*-isomer of the photoswitch. The measured Azo-SO_3_H concentrations are shown in Supplementary Table S2 as well as the resulting *P*_OW_ and log(*P*_OW_) values. Sample Nr. 6, 9 and 12 show an unusual high deviation from the other relevant measurements and were excluded from further data processing. The overall mean *P*_OW_ value for the samples 1–6) is 8.02 with a standard deviation of ±2.74. For samples 7–12) *P*_OW_ value of 3.32 ± 0.57 were calculated.

### Azo-SO_3_H partitioning into RBC membrane

To quantify the different partitioning of the isomers of Azo-SO_3_H between RBC membrane and the medium, the amount of photoswitch of a solution was measured spectroscopically via UV/vis absorbance before addition of RBCs and after extraction of the cells. For this, two solutions of Azo-SO_3_H (1 mL, 147 µM) in HEPES (300 mM, pH 7.0) were initially irradiated with vis light (30 s) to reach the PSS_*Z→E*_ and considered as the initial state. One of these solutions was consequently irradiated with UV light (30 s) to reach the PSS_*E→Z*_ and handled only under red light. To both solutions a constant amount of RBC containing medium (1 µL) was added and carefully mixed for about 2 min to yield an approximate RBC concentration of ~ 10^7 ^mL^-1^. To remove the RBCs and to yield a homogenous solution, the batches were then centrifuged (5 min, 100 g) and the supernatant additionally filtered through a syringe filter (Ø 15 mm, RC-membrane, 0.2 µm, Rotilabo®). Before measuring UV/vis spectroscopy both samples were irradiated with vis light (465 nm, 1 min) again to achieve the PSS_*Z*→*E*_. These experiments were repeated two times (see Supplementary Fig. 7). The difference between the initial and the spectra after RBCs addition and removal are shown by arrows. Using a linear calibration, the concentration of all the samples can be calculated and therefore the difference in Azo-SO_3_H concentration.

### Analysis of free-floating RBCs

To quantitatively determine the switching times of free-floating RBCs the acquired videos were analyzed by hand. In this procedure, the time between the first visible movement of the RBCs and reaching the equilibrium state where no obvious change was visible was measured. As this was done by hand the acquired data is biased and therefore can only be used to give an estimation of the two different switching times *t*_UV_ and *t*_vis_ for free-floating RBCs. To quantify the roughness of the RBC, a circle with a radius of 60 µm was drawn in the RBC (thus avoiding the boundaries) and the standard deviation of all pixel values inside this circle was calculated.

### Statistics

The scipy.stats package in Python was used for statistical analysis. Student’s independent *t* test was used for the evaluation of statistical significance. Three asterisks indicate a *p*-value less than 0.001.

### Micropipette aspiration

Self-made micropipettes were micro-forged from pulled glass-tubes and used for micropipette aspiration experiments. The inner diameter of the tip was made to have a size of ~2.1 µm. The experiments were carried out in open petri dishes. At first a passivation solution containing Casein (5 mg mL^−1^) was filled into the dish. Then the micropipette was loaded with the buffer that was later used in the experiment and dropped into the passivation solution for at least an hour. The casein solution was discarded and replaced by 2 mL of the buffer. Following, 1 µL of centrifuged RBCs were gently added to the buffer solution. The solution was homogenized by gentle mixing and the aspiration experiments were started immediately after the cells settled down. All measurements were repeated on at least 3 different days.

### Micropipette data analysis

Data analysis was performed with a home-written Python script^80^. Briefly, in order to analyze the time series, the images were first cropped and a square region of interest is drawn around the membrane interface in the micropipette, where the width of the region of interest is about half the width of the micropipette and its length long enough such that the minimum and maximum positions of the interface always fall within the square. The pixel intensities are then averaged over the width of the square to reduce noise, reducing the square to a line that crosses the membrane. Then the first derivative of the intensity with respect to the length along the pipette is taken to more reliably track the RBC membrane. The position of the membrane interface is then determined with sub-pixel resolution by fitting a second order polynomial to the minimum of the derivative. This procedure is repeated for the entire time series to obtain the position of the membrane as a function of time, an example of which can be seen in Fig. 3b in the main text.

### Surface area analysis

The change in penetration depth of the membrane in the micropipette relates to the total change in membrane area as follows. First, the RBC is split up in three sections: (I) a half-sphere cap on the end of the RBC in the capillary, (II) a cylindrical section of the RBC in the capillary, and (III) a sphere with a spherical cap removed that is made up by the RBC outside the capillary (see Supplementary Fig. 13).

The volume of these sections is as follows:

1 $${V}_{I}=\frac{2}{3}\pi {a}^{3}$$

2 $${V}_{{II}}=\pi {a}^{2}l$$

3 $${V}_{{III}}=\frac{\pi }{3}\left(2{r}^{3}+\left(2{r}^{2}+{a}^{2}\right)\sqrt{{r}^{2}-{a}^{2}}\right)$$

And their surface area is:

4 $${A}_{I}=2\pi {a}^{2}$$

5 $${A}_{{II}}=2\pi a\,l$$

6 $${A}_{{III}}=2\pi r\left(r+\sqrt{{r}^{2}-{a}^{2}}\right)$$

with *a* the micropipette radius, *r* the RBC radius and *l* the length of the RBC in the micropipette. Although in principle all three parameters *a*, *r*, and *l* can be extracted from the images, this would introduce an inaccuracy since the resolution of the images is relatively low. As the micropipette radius can be determined most accurately of the three parameters, we choose to calculate the radius of the RBC and the length of the cylindrical section, using the known volume (90 fL) and surface area (136 µm^2^) of RBC’s. This leads to two equations with two unknowns, which we solve numerically:

7 $${V}_{I}+{V}_{{II}}\left(l\right)+{V}_{{III}}\left(r,l\right)=90$$

8 $${A}_{I}+{A}_{{II}}\left(l\right)+{A}_{{III}}(r,l)=136$$

Without this approach, the values for *r*, *l* that are extracted from the images are close to the calculated values, yet the extracted values seem to structurally overestimate *r*, potentially due to the RBC being partly out of focus, and underestimate *l*, as the actual micropipette is not a perfect cylinder but tends to narrow near the tip.

As the surface area of the RBC changes upon illumination with either UV or visible light, the position of the membrane interface in the micropipette changes by a length *δl*. Additionally, as the total volume of the RBC remains constant but the volume residing in the micropipette changes, the volume and therefore the radius of the RBC outside the micropipette also changes. As this change in radius is small (in the order of one pixel in the images), instead of extracting the changed radius from the images, we calculate the change in radius using the fact that although the surface area changes, the RBC volume remains constant. Hence,

9 $${V}_{{III}}=\pi {a}^{2}\delta l+{V}_{{III},{new}}$$

In this equation the only unknown is the new RBC radius ${r}_{{new}}$. Obtaining ${r}_{{new}}$ from images is consistent with this approach, although the uncertainty higher. When ${r}_{{new}}$ is known, the surface area after the switch in illumination is calculated, with the following alterations:

10 $${A}_{{II},{new}}=2\pi a\,(l+\delta l)$$

11 $${A}_{{III},{new}}=2\pi r\left({r}_{{new}}+\sqrt{{{r}_{{new}}}^{2}-{a}^{2}}\right)$$

By subtracting the original RBC surface area (136 µm^2^) and dividing by the original RBC surface area, the relative change in surface area is obtained.

### Switching time analysis

The switching time is obtained by fitting an exponential function of the form

12 $$y\left(t\right)=a+b\,{e}^{-t/\tau }$$

to the RBC interface within the micropipette as a function of time, where τ is the characteristic switching time.

### Calculation of suction pressure and performed work

The suction pressure Δ*P* that can be applied with a micropipette can be calculated from density of water ρ, the gravitational acceleration g and the water reservoir displacement Δ*h:*

13 $$\Delta P=\rho g\Delta h$$

The used height adjustment has a mean value of Δ*h* = 979 µm giving an average suction pressure of Δ*P* = 9.604 pN/µm^2^, or an applied tension of ${10}^{-5}{{{{{\rm{N}}}}}}/{{{{{\rm{m}}}}}}$.

Together with the inner diameter of the micropipette the surface area *A* and the movement of the membrane Δ*d* inside the pipet the work *W* can be calculated:

14 $$W=A\Delta d\Delta P$$

With a mean surface area inside the pipet of *A* = 3.52 ± 0.06 µm^2^ and an average membrane movement of Δ*d* = 0.82 ± 0.30 µm the typical work was found to be *W* = 27.7 ± 10.1 aJ. Conversion to *k*_B_*T* using: *k*_B_*T* = 4.11 × 10^−21^ J gave *W* = 6.747 × 10^3^ ± 2.462 × 10^3^ *k*_B_*T*.

### Autocorrelation

The procedure to quantify the degree of change visible in the brightfield movies when cells are subject to UV/vis illumination in the presence of Azo-SO_3_H is as follows. First the Image Stabilizer plugin in ImageJ is used to correct a 2D image stack for drift in the horizontal plane^81^. Next, an area is cropped around a part of the cell membrane where movement upon illumination changes is visible by eye. Using a custom-written Python script, the first image of the stack is autocorrelated with every subsequent image. Here, in addition to sharp jumps that are a consequence to UV/vis switches, there is a general decay in the autocorrelation due to focus drift and (intra)cellular movement. To focus on the changes in the autocorrelation due to UV/vis switches, we remove this decay by fitting an exponential function of the form

15 $${{{{{\rm{y}}}}}}\left({{{{{\rm{t}}}}}}\right)=a{e}^{-t/\tau }+b$$

to the autocorrelation and subtracting the fitted function.

### Reporting summary

Further information on research design is available in the Nature Portfolio Reporting Summary linked to this article.

## Supplementary information

Supplementary Information

Description of Additional Supplementary Files

Supplementary Movie 1

Supplementary Movie 2

Supplementary Movie 3

Supplementary Movie 4

Supplementary Movie 5

Supplementary Movie 6

Supplementary Movie 7

Reporting Summary

## Data Availability

The data that support the findings of this study are available from the corresponding author upon request.

## Code availability

The code used in this study is available on [10.5281/zenodo.7908543] or from the corresponding author upon reasonable request.
