# Supplementary material for: Rapid and reversible optical switching of cell membrane area by an amphiphilic azobenzene
Source: Nat Commun. 2023 Jun 23;14:3760. doi: 10.1038/s41467-023-39032-0 (PMC10290115; doi:10.1038/s41467-023-39032-0)
Supplement: Supplementary file 1 — Supplementary Information [file 41467_2023_39032_MOESM1_ESM.pdf]

Supplementary Information

Rapid and reversible optical switching of cell membrane area by an amphiphilic azobenzene

Fabian Höglsperger[1,2], Bart E. Vos[3], Arne Hofemeier[3], Maximilian D. Seyfried[1,2], Bastian Stövesand[1,2], Azadeh Alavizargar[4], Leon Topp[4], Andreas Heuer[2,4], Timo Betz[3], Bart Jan Ravoo[1,2]**

1 Organic Chemistry Institute
University of Münster
Corrensstr. 36, 48149 Münster, Germany

2 Center for Soft Nanoscience
University of Münster
Busso-Peus-Str. 10, 48149 Münster, Germany

3 Third Institute of Physics—Biophysics
University of Göttingen
Friedrich-Hund-Platz 1, 37077 Göttingen, Germany

4 Institute of Physical Chemistry
University of Münster
Corrensstr. 28-30, Münster 48149, Germany

E-mail: b.j.ravoo@uni-muenster.de, timo.betz@phys.uni-goettingen.de

Table of contents

1 Instrumentation 2
2 Supplementary data 4
3 Synthesis 19
4 Simulations 22
5 References 24

1 Instrumentation

UV/vis spectroscopy

UV/vis spectra were measured on a *Jasco V-770* spectrophotometer (Jasco Deutschland GmbH, Pfungstadt, Germany) using *High Precision* quartz glass cuvettes (Hellma Analytics GmbH, Müllheim, Germany). The spectra were recorded with *Spectra Manager 2, Spectra Manager Version 2.14.06* (Jasco Deutschland GmbH, Pfungstadt, Germany). The samples were dissolved in the specified solvent and the baseline was measured against the same solvent. Data analysis was realized using *OriginPro 2018 b b9.5.5.409* (ORIGINLAB Corporation, Northampton, USA). If not otherwise stated the *Z*-isomer was handled in the dark under red light.

Photoisomerization

For *E*-to-*Z* photoisomerization a UV LED Gen2 Emitter (LZ1-00UV00) (365 nm) from LEDENGIN was used and for Z-to-*E* photoisomerization, a Philips Lumileds Royal Blue LUXEON K2 emitter (LXK2-PB14-Q00) emitting light at a wavelength of 465 nm was used.

Microscopy

Visualization of vesicles was achieved with a Nikon *Eclipse Ti-E Inverted Microscope system* equipped with *60 x water immersion objective* (Nikon Instruments Europe B.V., Germany). A 50 W halogen lamp was used for brightfield illumination. As light source for photoswitching and fluorescence microscopy was a four wavelength LED source (Thorlabs, Munich Germany) used. It was equipped with the following LEDs: 365 nm (85 mW), 490 nm (50 mW), 530 nm (100 mW) and 625 nm (250 mW). In a typical experiment the 365 nm LED to was set to 2% intensity corresponding to 117 μW and the 490 nm LED was set to 100% intensity which corresponds to 322 μW. Switching of the light sources and recording of the images was performed with a custom written program in LabView (National Instruments, Austin, USA). Images were taken with a *PCO sCMOS Camera* (PCO AG, Germany) at 12.5Hz. Images were analyzed and processed using *ImageJ version 1.52p* (National Institutes of Health, USA).

Mass spectroscopy

Electrospray ionization (ESI) mass spectra were recorded on a Bruker Daltonics *MicroTof* (Bruker Corporation, Billerica, Massachusetts, USA) or a Thermo Scientific *Orbitrap LTQ XL* (Thermo Fisher Scientific Inc., Waltham, Massachusetts, USA).

Nuclear magnetic resonance spectroscopy

The NMR spectra were recorded on a *Bruker AV300* and *AV400* (Bruker Corporation, Billerica, Massachusetts, USA) and on an *Agilent DD2 500* (Agilent Technologies Deutschland GmbH, Waldbronn, Germany). The measurements were performed at room temperature in the indicated deuterated solvents. The spectra were analyzed using the software *MestReNova 12.0.0-20080* (Mestrelab Research S. L., Santiago de Compostela, Spain). The chemical shifts (δ) are expressed in parts per million (ppm) relative to the residual solvent signals. The multiplicity of the signals is designated as *s* (singlet), *d* (doublet), *t* (triplet), *q* (quartet), *m* (multiplet) and *br* (broad). ^{1}H coupling constants *J* are given in Hz. ^{1}H NMR chemical shifts are given relative to TMS and are referenced to the solvent signal. Spectra of other nuclei such as ^{13}C are referenced according to the proton resonance of TMS as the primary reference for the unified chemical shift scale (IUPAC recommendation 2001).

2 Supplementary data

Photoisomerization of Azo-SO_3H

The half-life time $t_{1/2}$ of the Z-Azo-SO_3H was determined by using UV/vis spectroscopy. A solution of Azo-SO_3H in H_2O (c = 100 µM) was irradiated with UV light (365 nm) for 5 min. Spectra were recorded after different times and the absorbance at 346 nm was plotted against the time. Fitting the resulting data points yielded an exponential equation for calculating $t_{1/2}$. For the determination of $t_{1/2}$, the equations were solved for y = abs.@ $t_{1/2}$. The value of abs.@ $t_{1/2}$ was calculated using the following equation: $abs.@t_{1/2} = \frac{abs.@t0+abs.@t\infty}{2}$. This procedure was repeated for T = 50 °C, T = 60 °C and T = 70 °C. The obtained $t_{1/2}$ plotted against the temperature in a logarithmic plot and the $t_{1/2}$ at 25 °C was extrapolated from a linear fit.

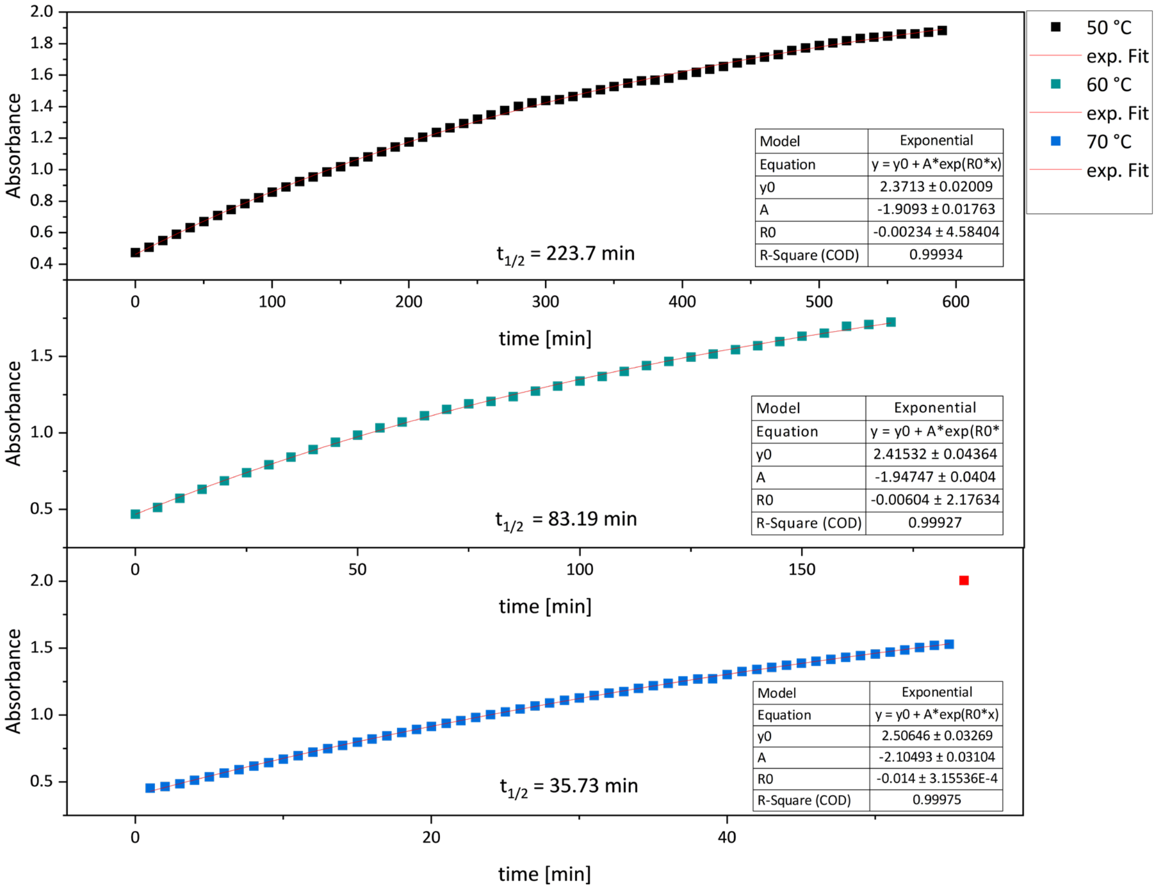

Supplementary Figure 1. Determination of the thermal half-life time of Azo-SO_3H (c = 100 µM in water) at T = 50 °C, T = 60 °C and T = 70 °C.

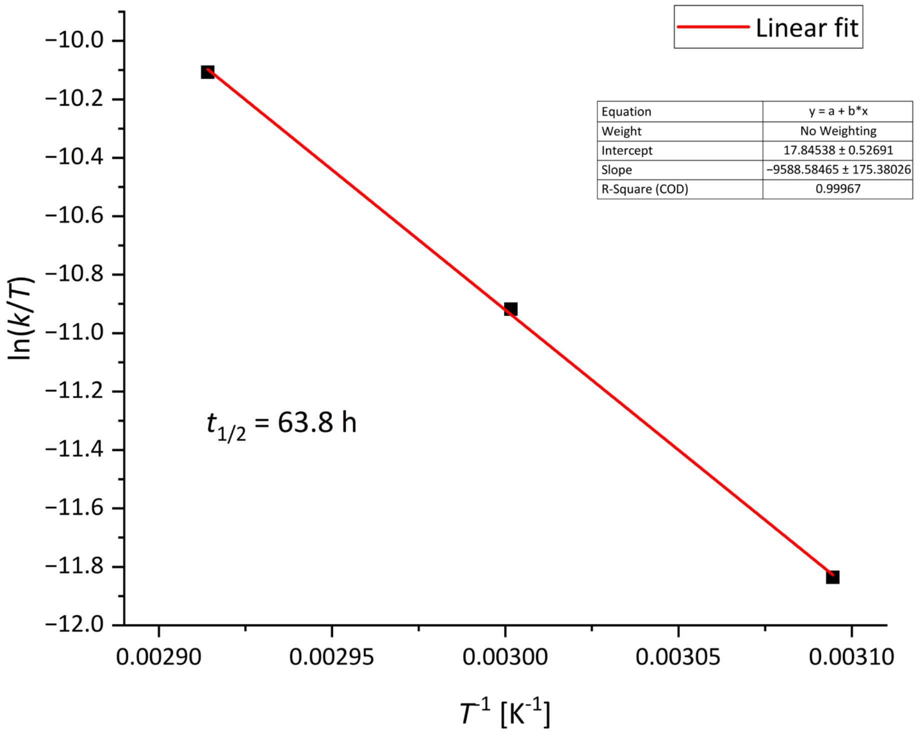

Supplementary Figure 2. Extrapolation of the thermal half-life time of Azo-SO_3H at room temperature (T = 25 °C).

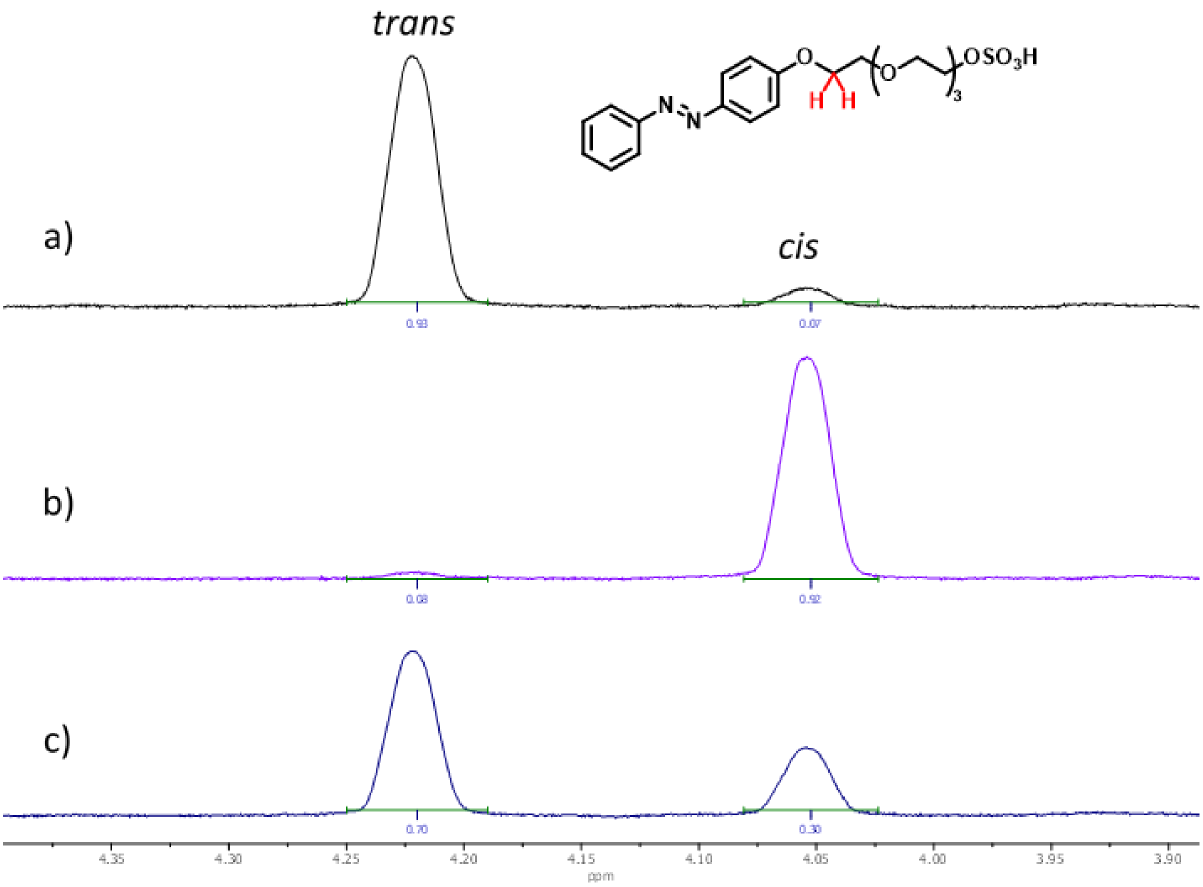

Supplementary Figure 3. Determination of the photostationary state (PSS) of Azo-SO_3H in DMSO-d6 (c = 2.5 mM). NMR spectra a) as synthesized; b) irradiated with UV light (365 nm) for 5 min c) irradiated with blue light (465 nm) for 5 min). From the peak integrals in the NMR spectra, $PSS_{E \rightarrow Z}$ = 92 % und $PSS_{Z \rightarrow E}$ = 70 %. Note that for NMR the concentration of the sample is higher than for all other experiments described in this study. The PSS may be affected by the inner filter effect of the sample.

Surface activity of Azo-SO3H

The dynamic changes in the surface tension were measured with a pendant drop tensiometer (PAT 1M Sinterface, Germany) where the sample compartment was equipped with an OG590 (Schott, Germany) bandpass filter to block wavelengths <590 nm from the light source of the tensiometer. This measure prevented unwanted switching of Azo-SO3H due to the instrument itself. The drop shape was analyzed using image analysis and the application of the Young-Laplace equation. Perpendicular to the optical bench of the tensiometer UV (365 nm) and blue (465 nm) LEDs were mounted on both sides of the pendant drop within the sample compartment. The samples were measured under continuous irradiation. The analysis of the surface tension isotherm was performed using the freely available IsoFit software. The CMC of *E*-Azo-SO3H was determined by linear extrapolation.

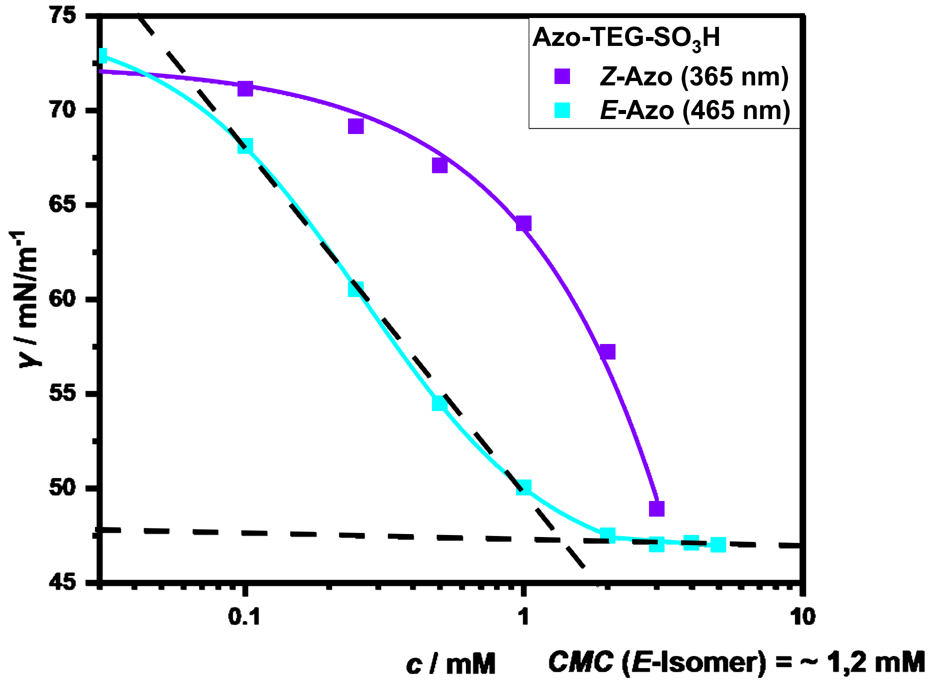

Supplementary Figure 4. Surface tension of a solution of Azo-SO3H in water under continuous irradiation with UV light (365 nm, purple curve) and blue light (465 nm, blue curve). The CMC for the *E*-isomer (blue curve) is 1.2 mM. The CMC for the *Z*-isomer can not be determined accurately due to poor PSS at higher concentration.

Partition coefficient of Azo-SO3H

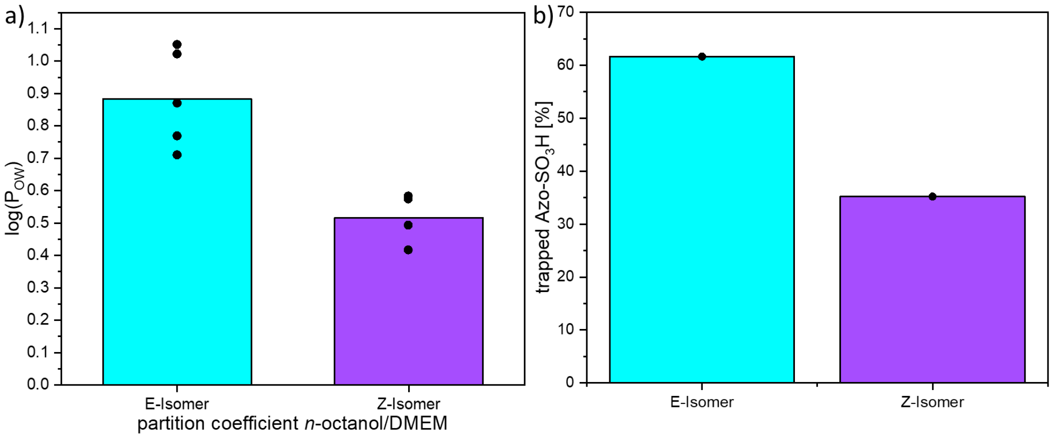

Supplementary Figure 5. a) Determination of the partition coefficient (n-octanol/DMEM) of *E*-Azo-SO_3H and *Z*-Azo-SO_3H (N = 1, n = 5 (*E*) or 4 (*Z*)). b) Trapping of Azo-SO_3H in the plasma membrane after addition of RBCs to a 147 µM solution of *E*-Azo-SO_3H or *Z*-Azo-SO_3H in HEPES (300 mM, pH 7.0) (N = 1, n = 3).

Supplementary Table 1. Composition of the experiments for the determination of the partition coefficient n-octanol/DMEM for AZO-SO_3H.

Sample	$V_{DMEM-stock}$ [mL	V_{DMEM} [mL]	$V_{n-octanol-stock}$ [mL]	$V_{n-octanol}$ [mL]
1)	2	1	0	3
2)	2	0	0	4
3)	2	2	0	2
4)	3	0	2	1
5)	4	0	2	0
6)	2	0	2	2
7)	2	1	0	3
8)	2	0	0	4
9)	2	2	0	2
10)	3	0	2	1
11)	4	0	2	0
12)	2	0	2	2

Supplementary Table 2. Sample concentration for both phases and resulting P_{OW} and $\log(P_{OW})$ values.

Sample	C_{DMEM} [µM]	$C_{n\text{-}Octanol}$ [µM]	P_{OW}	$\log(P_{OW})$
1)	18.14	106.84	5.89	0.77
2)	15.80	80.38	5.09	0.71
3)	14.99	157.53	10.51	1.02
4)	10.95	80.61	7.36	0.87
5)	10.69	120.05	11.23	1.05
6)	21.06	60.74	2.88	0.46
7)	27.17	101.84	3.75	0.57
8)	29.49	77.17	2.62	0.42
9)	27.52	147.72	5.37	0.73
10)	24.81	76.86	3.10	0.49
11)	29.20	111.83	3.83	0.58
12)	32.42	57.76	1.78	0.25

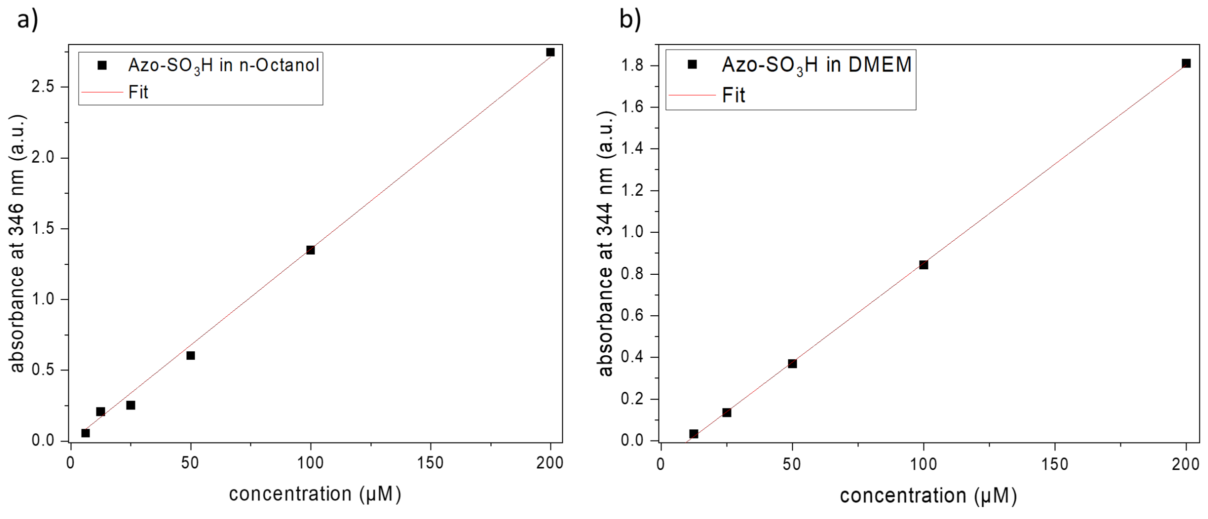

Supplementary Figure 6. UV/vis calibration for Azo-SO_3H in n-octanol a) and DMEM b) in a concentration range from 6.25 µM to 200 µM. Prior to measurement the samples were irradiated with vis light for 5 min.

Partitioning of Azo-SO_3H in RBCs

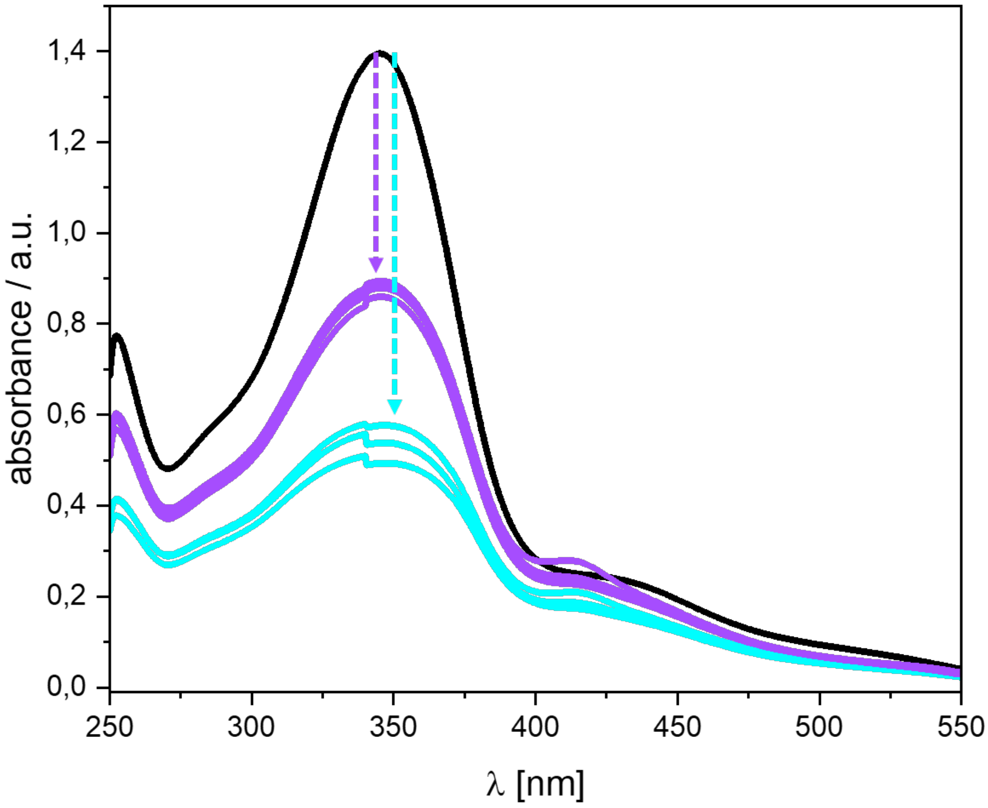

Supplementary Figure 7. UV/vis absorbance spectra to determine the difference in concentration of Azo-SO_3H in the supernatant HEPES buffer (300 mM, pH 7.0) before (black, c = 147 µM) and after the addition of RBCs to the *E*-isomer (cyan) and to the *Z*-isomer (violet). Note that after removal of the RBCs, the supernatant was irradiated with blue light to obtain *E*-isomer in each case. The arrows point out the reduction of absorbance due to partitioning into the RBC.

Photomanipulation of RBCs

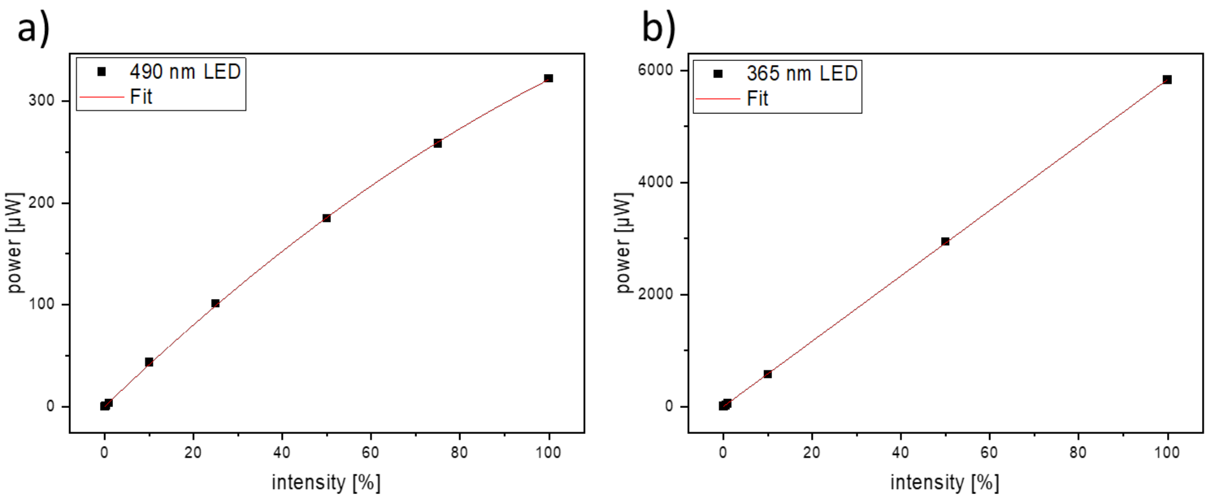

Supplementary Figure 8. (a-b) Illumination power of the used LEDs on the microscope vs. intensity.

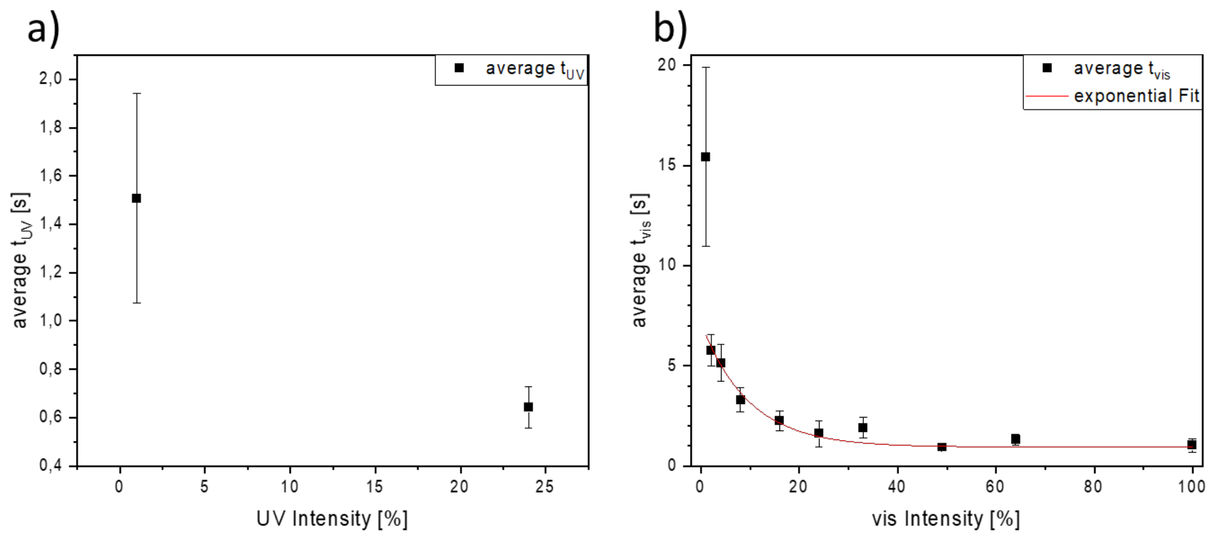

Supplementary Figure 9. Average switching times t_{UV} (a) and t_{vis} (b) in correlation to irradiation intensity for free-floating RBCs incorporated with Azo-SO_3H (c = 1 mM) in DMEM. (N = 1, n = 3.) Error bars show the standard deviation.

Supplementary Table 3. Average switching times t_{UV} for different UV intensities.

UV intensity [%]	average t_{UV} [s]	standard deviation [s]
1	1.508	0.435
24	0.642	0.086

Supplementary Table 4. Average switching times t_{vis} for different vis intensities.

vis intensity [%]	average t_{vis} [s]	standard deviation [s]
1	15.430	4.453
2	5.770	0.804
4	5.146	0.902
8	3.288	0.606
16	2.263	0.493
24	1.607	0.653
33	1.909	0.509
49	0.927	0.073
64	1.321	0.266
100	1.021	0.324

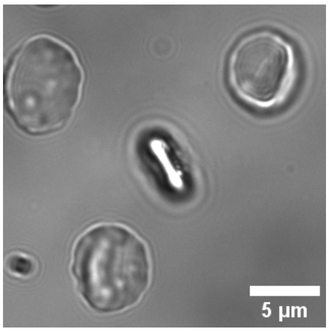

Supplementary Figure 10. Brightfield microscopy image of a free-floating RBC in the discocyte-like state. The side view confirms that the RBC is indeed biconcave and hence discocyte-like, and not oblate. N = 1, n > 10.

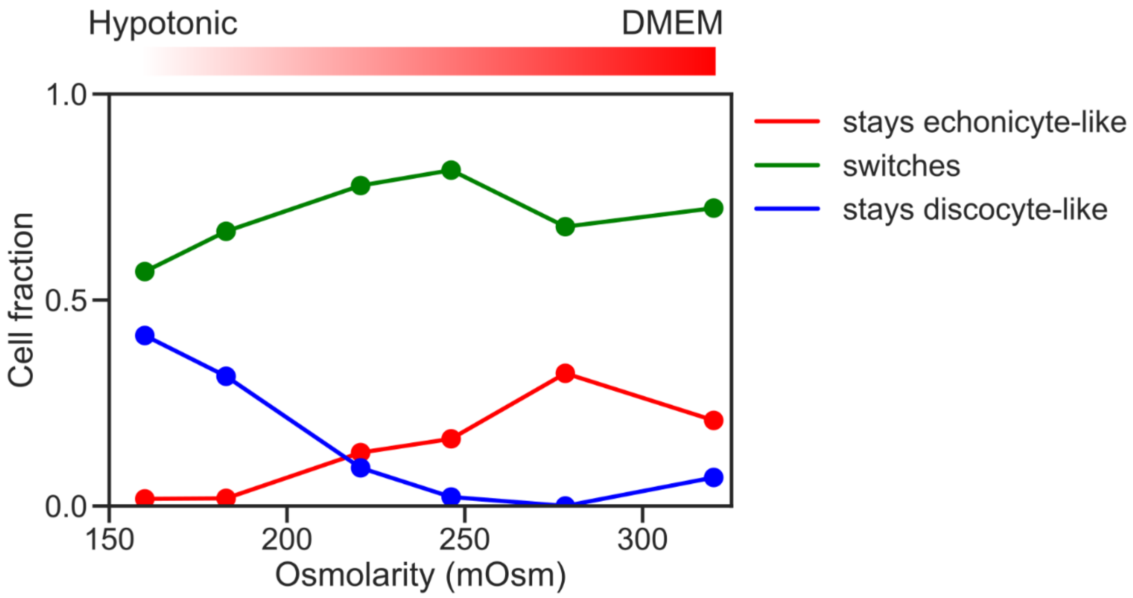

Supplementary Figure 11. Confirming that some RBCs do not switch between echinocyte-like and discocyte-like shape due to a changed volume/surface area ratio generated by decreasing the osmolality. The graph shows the fraction of RBCs that, in the presence of Azo-SO_3H and with switching UV/vis illumination, switches between echinocyte-like and discocyte-like shape, stays in the echinocyte-like shape or stays in the discocyte-like shape, as a function of the fraction of distilled water in the medium. By increasing this fraction (and thereby decreasing the salt concentration in the medium) the RBCs swell and change their volume/surface area ratio. Between 54 and 130 RBCs were counted for each condition of the medium.

Micropipette experiments

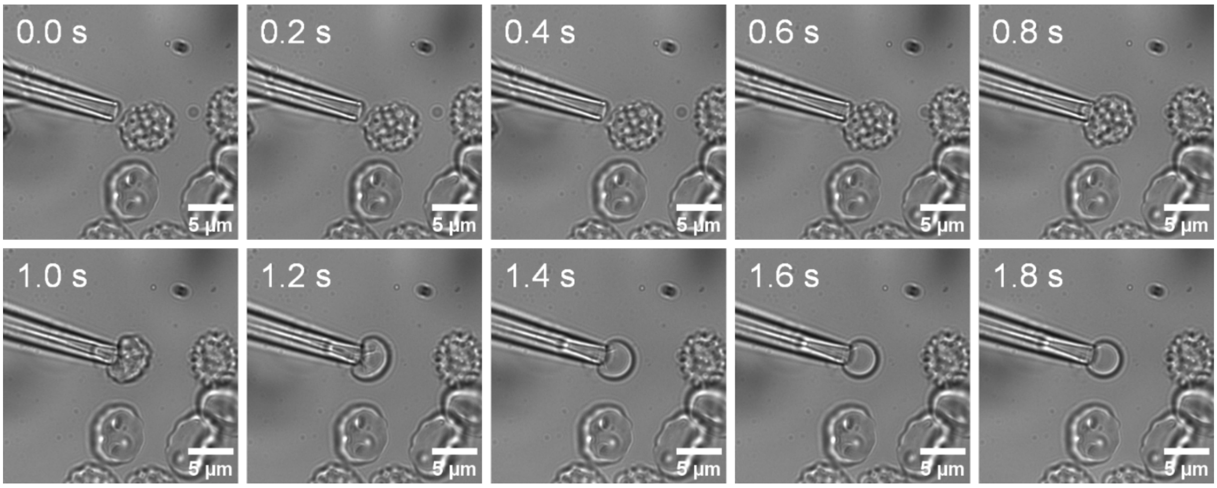

Supplementary Figure 12. Snapshots of an echinocyte RBC aspirated onto a micropipette. The echinocyte transforms to a discocyte upon aspiration.

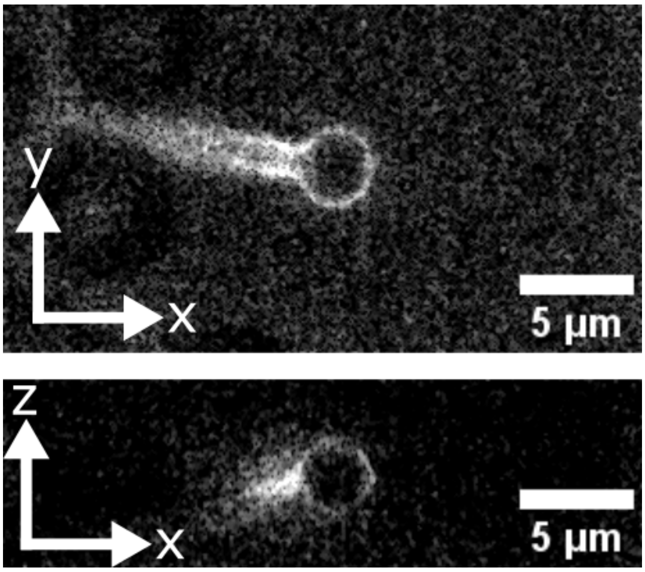

Supplementary Figure 13. Fluorescence microscopy of aspirated RBCs labeled with ATTO 488. Comparison of the xy and xz planes indicates that the RBC is spherical and the aspirated volume in the pipette is cylindrical. In order to reconstruct the xz-plane, a 3D stack with z = 0.25 µm separation between the slices was taken. Each frame was illuminated for 200 ms with 490 nm light. Reconstruction of the xz-plane was done using FIJI. N = 1, n = 3.

Control experiments

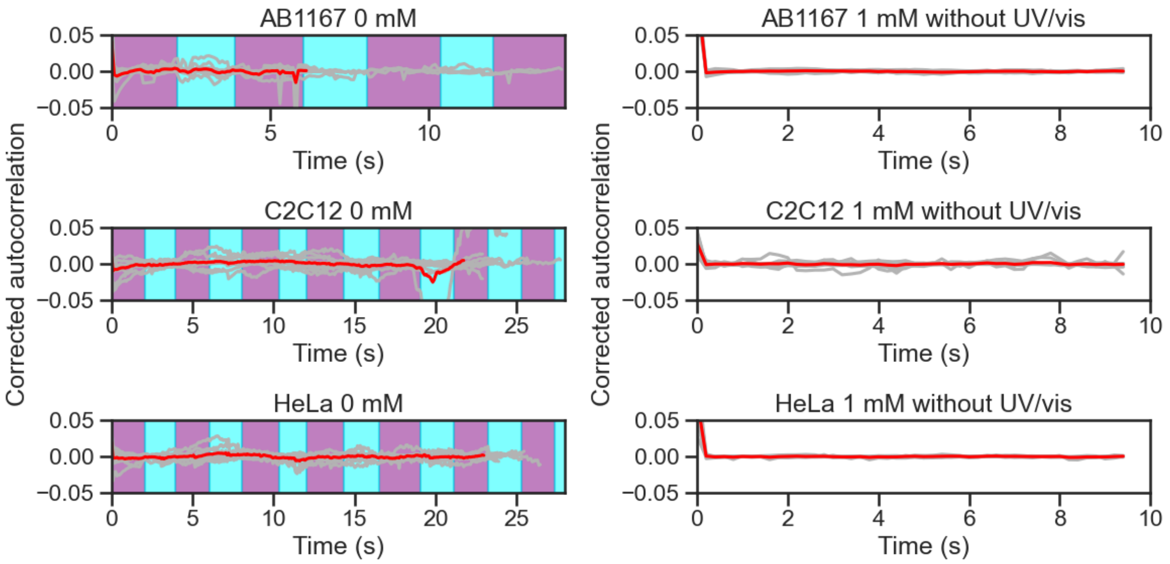

Supplementary Figure 14. Control experiments showing the corrected autocorrelation for cells under switching UV/vis illumination in the absence of Azo-SO_3H, and cells with 1 mM Azo-SO_3H but without switching light conditions.

Cell viability studies

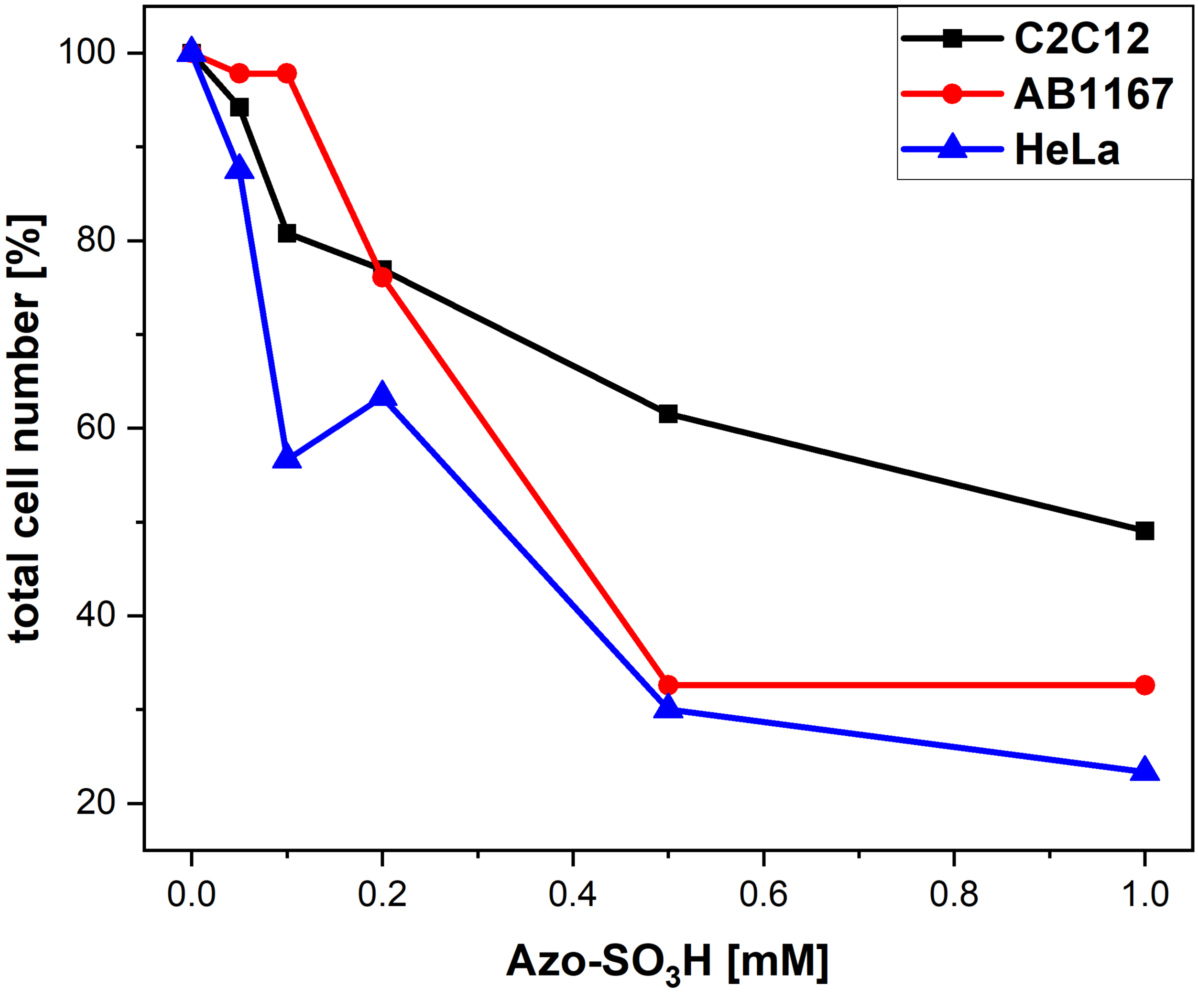

Supplementary Figure 15. Total cell count depending on the concentration of Azo-SO_3H.

C2C12

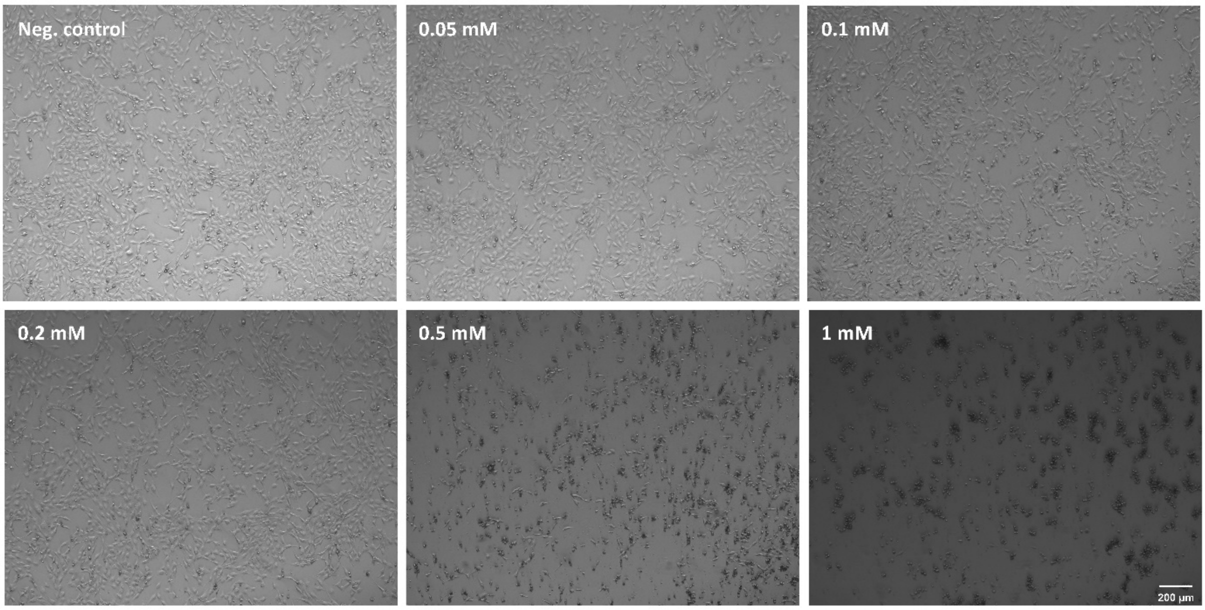

Supplementary Figure 16. Viability assay for murine C2C12 myoblast cells with Azo-SO_3H (c = 0.0-1.0 mM) after 24 hours. N = 1, n > 255,000.

AB1167

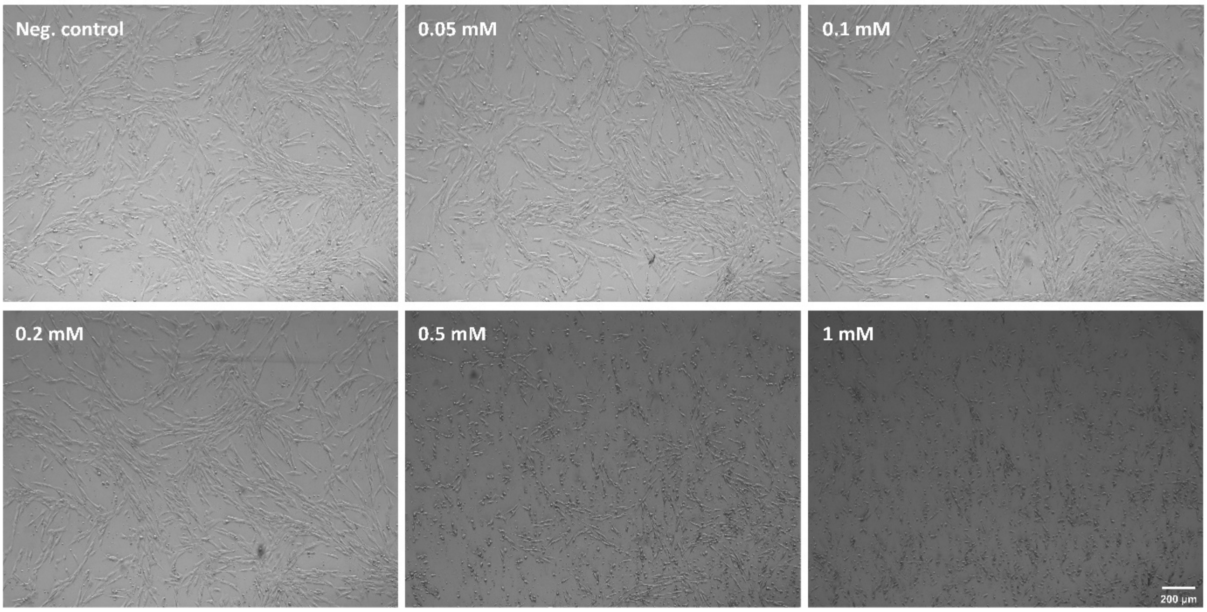

Supplementary Figure 17. Viability assay for human AB1167 myoblast cells with Azo-SO_3H (c = 0.0-1.0 mM) after 24 hours. N = 1, n > 30,000.

HeLa

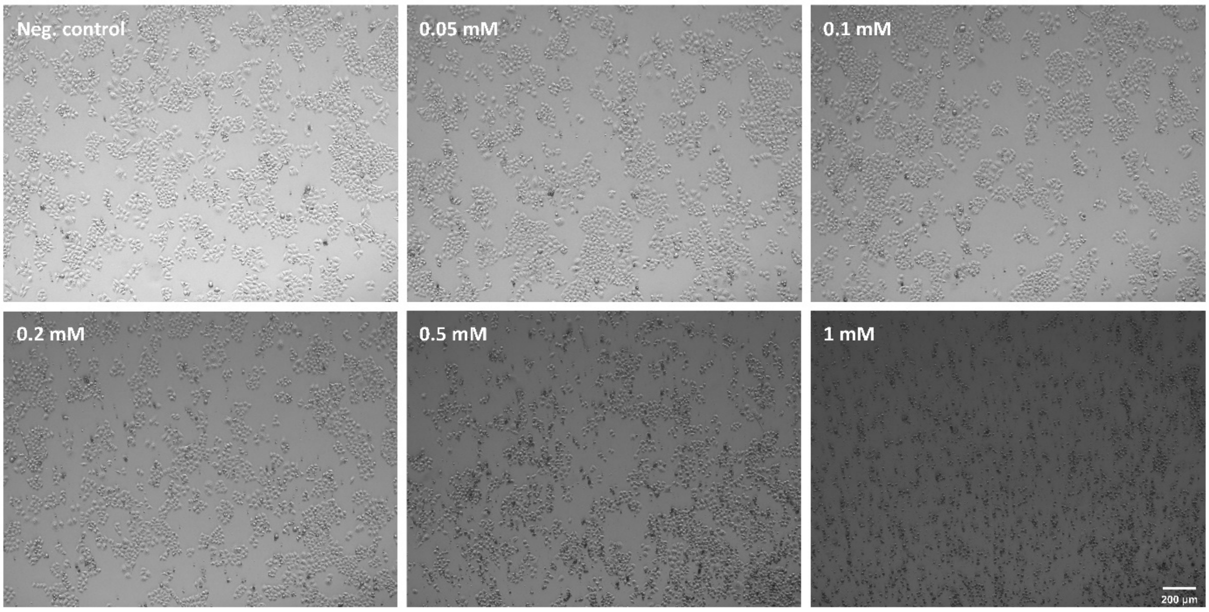

Supplementary Figure 18. Viability assay for human HeLa cervical cells with Azo-SO_3H (c = 0.0-1.0 mM) after 24 hours. N = 1, n > 140,000.

3 Synthesis

General

Moisture or air sensitive reactions were carried out under inert Argon atmosphere. For this oven-dried and vacuum-heated glassware and standard SCHLENK technique was used. Solvents were dried over molecular sieves for at least overnight.

Acetonitrile over 3 Å molecular sieves

Dichlormethane over 4 Å molecular sieves

N′,*N′*-Dimethylformamide over 4 Å molecular sieves

Thin layer chromatography (TLC) was performed on *Silica gel 60 F254* coated aluminium foils bearing a fluorescence indicator (Merck KGaA, Darmstadt). Spots were analyzed by irradiation under UV light (254 nm) and by dipping into basic KMnO4-solution and drying. Purification via preparative silica gel column chromatography was performed using Silica Gel (Merck KGaA, Darmstadt, Germany) with a grain size of 40 – 65 µm. Automated preparative chromatography was performed on Reveleris X2 flash chromatography system (Büchi Labortechnik, Flawil, Switzerland) equipped with a Flash Pure Ecoxflex 12 g C 18 (Büchi Labortechnik, Flawil, Switzerland) reverse phase column. Signals were detected on an UV and an evaporative light scattering (ELSD) detector. Ultrapure water with an electrical resistance higher than 18 MΩ was prepared with an *ELGA Purelab flex 3* (ELGA LabWater, High Wycombe) water purification system. Lyophilization was performed using an *Alpha 1-2 LD plus freeze dryer* (Martin Christ GmbH, Osterode, Germany). Prior to lyophilization all substances were dissolved in ultrapure water and frozen under rotation in liquid nitrogen. As syringe filters Rotilabo® Mini-Tip syringe filter, Ø 15 mm, RC-membrane, 0.45 µm (Carl Roth GmbH+Co. KG, Karlsruhe, Germany) were used. Sulfur pyridine complex was bought from *TCI* (TCI Co., Ltd., Tokyo, Japan) and used without further purification. (*E*)-2-(2-(2-(2-(4-(phenyldiazenyl)phenoxy)ethoxy)ethoxy)ethoxy)ethan-1-ol was synthesized according to a previously reported procedure.[1]

Synthesis of Azo-SO3H

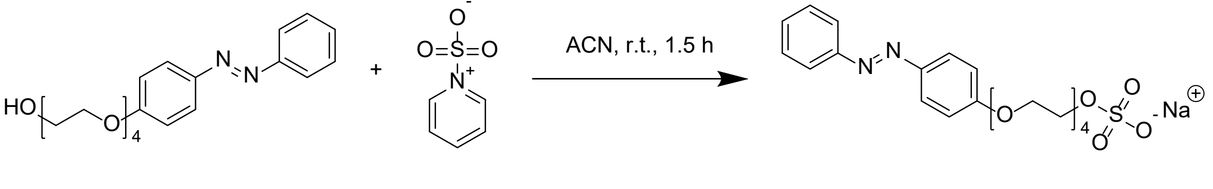

(*E*)-2-(2-(2-(2-(4-(phenyldiazenyl)phenoxy)ethoxy)ethoxy)ethoxy)ethan-1-ol (892.0 mg, 2.382 mmol, 1.0 eq) was dissolved in ACN (dry, 10 mL). Sulfur trioxide pyridine complex

(569.6 mg, 3.579 mmol, 1.5 eq) was added to the solution and stirred at r.t. for 1.5 h. To quench the reaction, NaOH (1M) was added until pH 11 was reached. Consequently, the solvent was removed under reduced pressure. The mixture was purified on a reverse phase column chromatography via automated preparative chromatography (1/9, ACN/H_2O, V/V→8/2, ACN/H_2O, V/V). The organic solvents were evaporated under reduced pressure and the remaining compound was lyophilized to yield a red oil.

Yield: 950.4 mg (1.996 mmol, 84%)

^{1}H-NMR (400 MHz, D_2O): δ = 7.37 (dd, J = 14.6, 8.0 Hz, 4H), 6.97 – 6.78 (m, 3H), 6.44 (d, J = 8.7 Hz, 2H), 4.01 – 3.95 (m, 2H), 3.59 – 3.43 (m, 4H), 3.41 – 3.16 (m, 10H).

^{13}C-NMR (101 MHz, D_2O): δ = 160.76, 151.91, 146.99, 146.26, 140.95, 130.31, 128.84, 127.27, 124.61, 122.34, 114.56, 71.63, 69.70, 69.50, 69.43, 68.76, 68.63, 67.36, 67.16, 60.31.

MS (m/z): (ESI, MeOH) Calculated for $[C_{20}H_{25}N_2O_8S_1Na_2]^+$: 499.11325, found 499.11240.

MS (m/z): (ESI, MeOH) Calculated for $[C_{20}H_{25}N_2O_8S_1]^-$: 453.13371, found 453.13291.

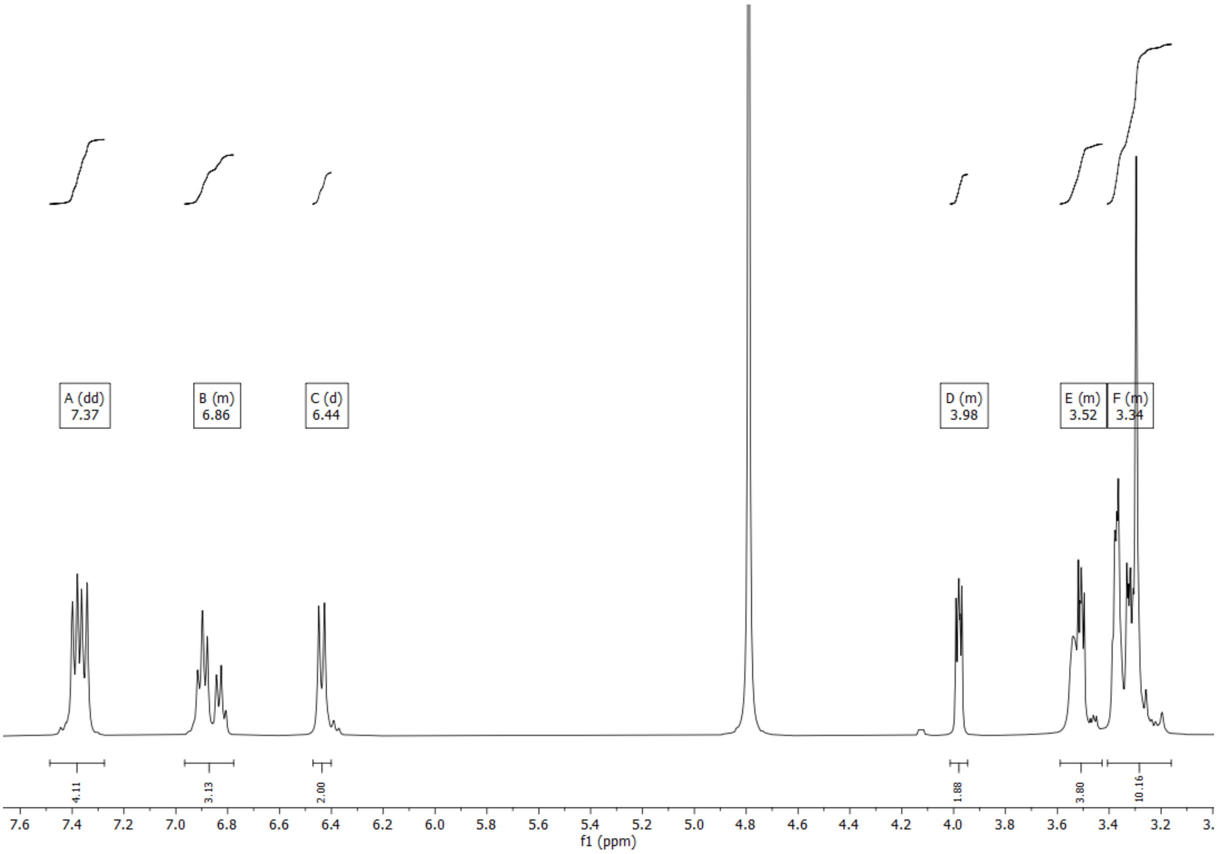

Supplementary Figure 19. ^{1}H-NMR spectrum of Azo-SO_3H in D_2O (Solvent signal at 4.79 ppm).

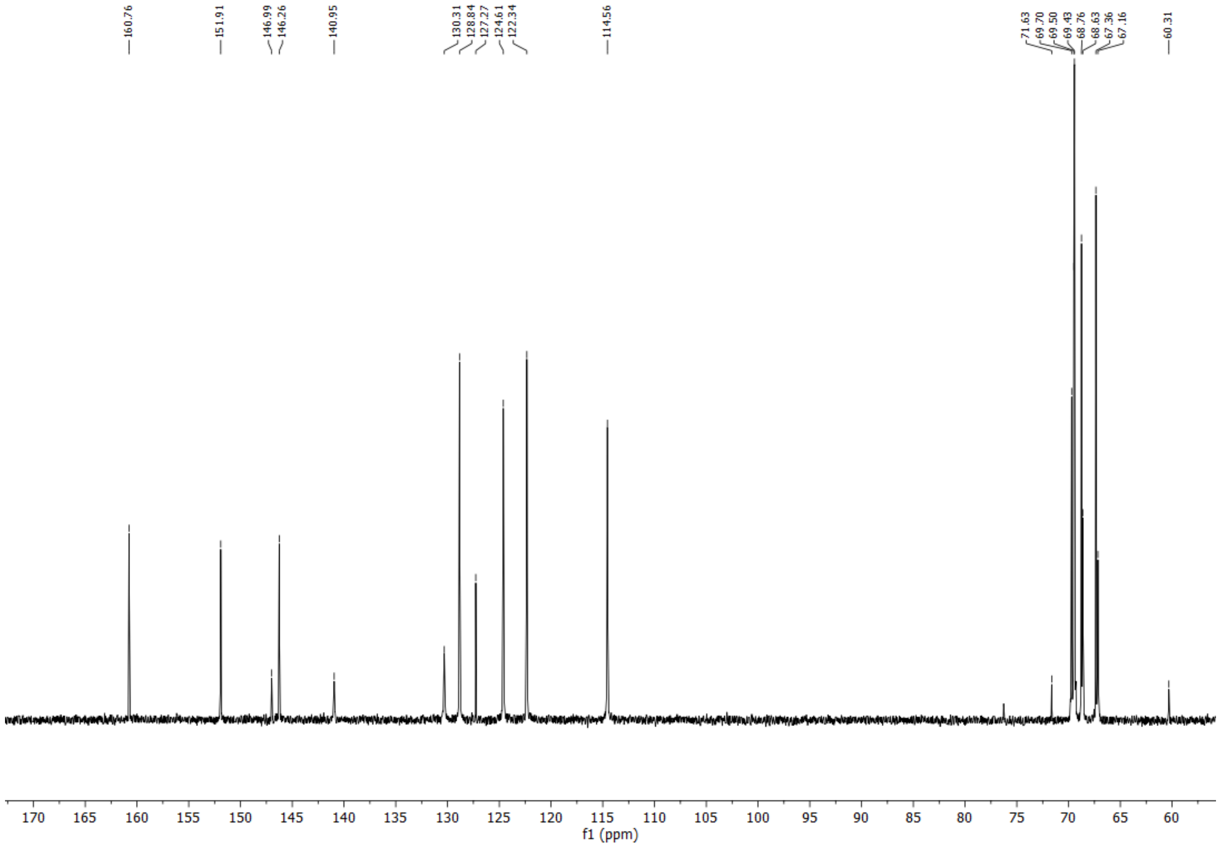

Supplementary Figure 20. ^{13}C-NMR spectrum of Azo-SO_3H in D_2O.

4 Simulations

System preparation

Force field parameters for the *E*- and *Z*-isomers of Azo-SO_3H were taken from the general Amber force field (GAFF).[2] For the calculation of the partial charges first the geometry of each isomer was optimized on the B3LYP/6-31G*[3] level of theory using Gaussian16.[4] The restrained electrostatic potential (RESP) charges were afterwards calculated using Antechamber[5] from the ESP charges obtained from a Hartree-Fock/6-31G* calculation.[3] Since the energy barrier between the *E*- and *Z*-isomers is too low as derived from the original GAFF, a modification of the corresponding dihedral angle potential was performed as described in the paper of Zheng et al.[6] Furthermore, the Lennard-Jones parameters for the hydrogen atom bound to the sulfur were set to $\varepsilon = 0.046$ kCal/mol and $\sigma = 4.0$ nm as used in the TIP3P water model as implemented in CHARMM.[7]

In the next step, a pure POPC membrane was constructed using the CHARMM-GUI membrane builder.[8] Next, two separate systems with the *Z*- or *E*-isomer and the membrane were constructed. For each system, Azo-SO_3H was inserted at a distance of 3 nm from the center of the membrane, and subsequently, water molecules were added to the system using GROMACS tools.

MD simulations

The MD simulations were performed using version 2019.6 of GROMACS,[9] and using the structure and parameter files obtained as described above. The TIP3P model was used for the water molecules. Periodic boundary conditions were applied in all directions. The long-range electrostatic interactions were treated using the particle mesh Ewald method, with a cutoff distance of 1.2 nm and a compressibility value of 4.5×10^{-5}.

For the van der Waals (vdW) interactions, cut-off schemes with a cutoff distance of 1.2 nm were used, which are smoothly truncated between 1.0 and 1.2 nm. Constant pressure at 1 bar was controlled by coupling the system to the Berendsen in equilibration and Parrinello-Rahman barostat in production simulations, using the semi-isotropic pressure scheme. The temperature was controlled at 310 K by coupling the system to the Nosé-Hoover thermostat.[3] The LINCS algorithm was employed to constrain the bonds.[10] The systems were first minimized and subsequently equilibrated using initially the NVT (500 ps) and then the NPT (16 ns) protocol in multiple steps. During the course of equilibration, restraints (1000 $kJ/mol^{-1}nm^{-2}$) were applied on Azo-SO_3H so that the membrane can be equilibrated before Azo-SO_3H interacts with it. The production simulations for both isomers were performed for 500 ns using a time step of 2 fs.

The simulation data were analyzed using GROMACS tools as well as in-house codes. VMD was used to visualize the structures and trajectories as well as to prepare the snapshots.[11]

MD simulations for truncated Azo-SO_3H

We additionally simulated truncated Azo-SO_3H without the ethylene glycol chain and the sulfate group (i.e. unsubstituted azobenzene, Azo) inside a POPC membrane to probe the effect of the chain on the behavior of the two isomers inside the membrane. The system preparation as well as the simulation parameters and procedure were similar to the Azo-SO_3H systems except that the two molecules of Azo were placed inside the membrane rather than in the solution.

Interestingly, the truncated Azo isomers without the ethylene glycol chain and the sulfate group show a very different behavior compared to the Azo-SO_3H isomers. Contrary to Azo-SO_3H, which remains only in one leaflet (see Figure 6), the truncated Azo isomers move regularly between the two leaflets, which can be concluded from the density plots (see Supplementary Figure 21). These movements are more frequent for the *E*-isomer likely due to the more hydrophobic nature of this isomer compared to the *Z*-isomer, and therefore, the density profile is more symmetric, whereas for the *Z*-isomer these movements are less frequent, which is why the density profile is rather more asymmetric.

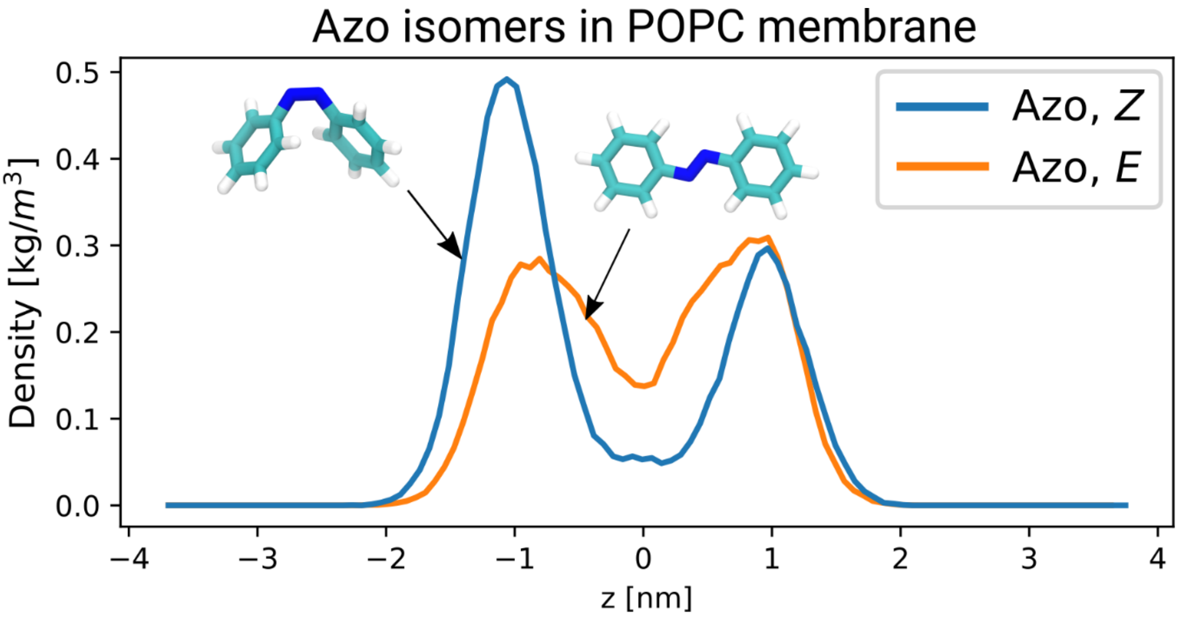

Supplementary Figure 21. The density profiles of truncated Azo isomers without the ethylene glycol chain and the sulfate group simulated inside a POPC membrane.

5 Supplementary References

(1) Moratz, J., Samanta, A., Voskuhl, J., Mohan Nalluri, S. K. & Ravoo, B. J. Light-triggered capture and release of DNA and proteins by host-guest binding and electrostatic interaction. *Chem. Eur. J.* **21,** 3271–3277 (2015).

(2) Wang, J.; Wolf, R. M.; Caldwell, J. W.; Kollman, P. A.; Case, D. A. Development and Testing of a General Amber Force Field. *J. Comput. Chem.* **2004**, *25* (9), 1157–1174. https://doi.org/https://doi.org/10.1002/jcc.20035.

(3) Ditchfield, R.; Hehre, W. J.; Pople, J. A. Self-Consistent Molecular-Orbital Methods. IX. An Extended Gaussian-Type Basis for Molecular-Orbital Studies of Organic Molecules. *J. Chem. Phys.* **1971**, *54*, 724–728. https://doi.org/10.1063/1.1674902.

(4) Frisch, M. J.; Trucks, G. W.; Schlegel, H. B.; Scuseria, G. E.; Robb, M. A.; Cheeseman, J. R.; Scalmani, G.; Barone, V.; Petersson, G. A.; Nakatsuji, H.; Li, X.; Caricato, M.; Marenich, A. V; Bloino, J.; Janesko, B. G.; Gomperts, R.; Mennucci, B.; Hratchian, H. P.; Ortiz, J. V; Izmaylov, A. F.; Sonnenberg, J. L.; Williams-Young, D.; Ding, F.; Lipparini, F.; Egidi, F.; Goings, J.; Peng, B.; Petrone, A.; Henderson, T.; Ranasinghe, D.; Zakrzewski, V. G.; Gao, J.; Rega, N.; Zheng, G.; Liang, W.; Hada, M.; Ehara, M.; Toyota, K.; Fukuda, R.; Hasegawa, J.; Ishida, M.; Nakajima, T.; Honda, Y.; Kitao, O.; Nakai, H.; Vreven, T.; Throssell, K.; Montgomery Jr., J. A.; Peralta, J. E.; Ogliaro, F.; Bearpark, M. J.; Heyd, J. J.; Brothers, E. N.; Kudin, K. N.; Staroverov, V. N.; Keith, T. A.; Kobayashi, R.; Normand, J.; Raghavachari, K.; Rendell, A. P.; Burant, J. C.; Iyengar, S. S.; Tomasi, J.; Cossi, M.; Millam, J. M.; Klene, M.; Adamo, C.; Cammi, R.; Ochterski, J. W.; Martin, R. L.; Morokuma, K.; Farkas, O.; Foresman, J. B.; Fox, D. J. Gaussian˜16 {R}evision {B}.01. 2016.

(5) Wang, J.; Wang, W.; Kollman, P. A.; Case, D. A. Automatic Atom Type and Bond Type Perception in Molecular Mechanical Calculations. *J. Mol. Graph. Model.* **2006**, *25* (2), 247–260. https://doi.org/https://doi.org/10.1016/j.jmgm.2005.12.005.

(6) Zheng, X.; Wang, D.; Shuai, Z.; Zhang, X. Molecular Dynamics Simulations of the Supramolecular Assembly between an Azobenzene-Containing Surfactant and α-Cyclodextrin: Role of Photoisomerization. *J. Phys. Chem. B* **2012**, *116* (2), 823–832. https://doi.org/10.1021/jp2073107.

(7) MacKerell, A. D.; Bashford, D.; Bellott, M.; Dunbrack, R. L.; Evanseck, J. D.; Field, M. J.; Fischer, S.; Gao, J.; Guo, H.; Ha, S.; Joseph-McCarthy, D.; Kuchnir, L.; Kuczera, K.; Lau, F. T. K.; Mattos, C.; Michnick, S.; Ngo, T.; Nguyen, D. T.; Prodhom, B.; Reiher, W. E.; Roux, B.; Schlenkrich, M.; Smith, J. C.; Stote, R.; Straub, J.; Watanabe, M.; Wiórkiewicz-Kuczera, J.; Yin, D.; Karplus, M. All-Atom Empirical Potential for Molecular Modeling and Dynamics Studies of Proteins. *J. Phys. Chem. B* **1998**, *102* (18), 3586–3616. https://doi.org/10.1021/jp973084f.

(8) Jo, S.; Kim, T.; Iyer, V. G.; Im, W. CHARMM-GUI: A Web-Based Graphical User Interface for CHARMM. *J. Comput. Chem.* **2008**, *29* (11), 1859–1865. https://doi.org/10.1002/jcc.20945.

(9) Lindahl, E.; Hess, B.; van der Spoel, D. GROMACS 3.0: A Package for Molecular Simulation and Trajectory Analysis. *Mol. Model. Annu.* **2001**, *7* (8), 306–317. https://doi.org/10.1007/s008940100045.

(10) Hess, B.; Bekker, H.; Berendsen, H. J. C.; Fraaije, J. G. E. M. LINCS: A Linear Constraint Solver for Molecular Simulations. *J. Comput. Chem.* **1997**, *18* (12), 1463–1472. https://doi.org/10.1002/(SICI)1096-987X(199709)18:12<1463::AID-JCC4>3.0.CO;2-H.

(11) Humphrey, W.; Dalke, A.; Schulten, K. VMD: Visual Molecular Dynamics. *J. Mol. Graph.* **1996**, *14* (1), 33–38. https://doi.org/http://dx.doi.org/10.1016/0263-7855(96)00018-5.
