# Supplementary material for: Rapid and reversible optical switching of cell membrane area by an amphiphilic azobenzene
Source: Nat Commun. 2023 Jun 23;14:3760. doi: 10.1038/s41467-023-39032-0 (PMC10290115; doi:10.1038/s41467-023-39032-0)
Supplement: Supplementary file 2 — Description of Additional Supplementary Files [file 41467_2023_39032_MOESM2_ESM.pdf]

Description of Additional Supplementary Files

File Name: Supplementary Movie 1
Description: Photomanipulation of free floating RBCs.

File Name: Supplementary Movie 2
Description: Photomanipulation of aspirated RBCs.

File Name: Supplementary Movie 3
Description: Addition of Azo-SO3H to RBCs.

File Name: Supplementary Movie 4
Description: Capture of RBC on micropipette.

File Name: Supplementary Movie 5
Description: Photomanipulation of C2C12 cells.

File Name: Supplementary Movie 6
Description: Photomanipulation of AB1167 cells.

File Name: Supplementary Movie 7
Description: Photomanipulation of HeLa cells.
